# Haploinsufficiency of GCP4 induces autophagy and leads to photoreceptor degeneration due to defective spindle assembly in retina

**DOI:** 10.1038/s41418-019-0371-0

**Published:** 2019-06-17

**Authors:** Zhigang Li, Huirong Li, Xu Xu, Lingling Wang, Bo Liu, Weixin Zheng, Lili Lian, Ying Song, Xizhong Xia, Ling Hou, Hanhua Cheng, Rongjia Zhou

**Affiliations:** 1 Hubei Key Laboratory of Cell Homeostasis, College of Life Sciences, Wuhan University, Wuhan, 430072 China; 2 State Key Laboratory of Opthalmology, Optometry and Vision Science, Wenzhou Medical University, Wenzhou, 325003 China

**Keywords:** Macroautophagy, Gene expression

## Abstract

Retinopathy, owing to damage to the retina, often causes vision impairment, and the underlying molecular mechanisms are largely unknown. Using a gene targeting strategy, we generated mice with the essential gene *Tubgcp4* knocked out. Homozygous mutation of *Tubgcp4* resulted in early embryonic lethality due to abnormal spindle assembly caused by GCP4 (gamma-tubulin complex protein 4, encoded by *Tubgcp4*) depletion. Heterozygotes were viable through dosage compensation of one wild-type allele. However, haploinsufficiency of GCP4 affected the assembly of γ-TuRCs (γ-tubulin ring complexes) and disrupted autophagy homeostasis in retina, thus leading to photoreceptor degeneration and retinopathy. Notably, GCP4 exerted autophagy inhibition by competing with ATG3 for interaction with ATG7, thus interfering with lipidation of LC3B. Our findings justify dosage effects of essential genes that compensate for null alleles in viability of mutant mice and uncover dosage-dependent roles of GCP4 in embryo development and retinal homeostasis. These data have also clinical implications in genetic counseling on embryonic lethality and in development of potential therapeutic targets associated with retinopathy.

## Introduction

In mammals, loss-of-function mutations often lead to early embryonic lethality, also a major cause of infertility. More than 50 million people globally have infertility and cannot access the essential interventions [1]. Despite viable pregnancies with embryos carrying gene mutations, a considerable number of newborns (~303,000) die within 4 weeks of birth each year worldwide, owing to congenital anomalies [2].

Considerable efforts have been made in screening for genes essential for cell survival in genomes. In mice, 489 genes have been knocked out by gene targeting in ES cells; 29% are lethal at postnatal day 14 and 13% are survivable (less homozygotes than predicted), whereas 58% are viable [3]. Multiplex RNAi screening has generated 166 important genes for growth in two human mammary cell lines [4]. Systematic pooled shRNA screening efforts have expanded to cancer cell lines [5–7]. A number of commonly essential genes and cell lineage-specific essential genes have been identified, thus facilitating identification of drivers of cancer cell growth and development of anti-cancer strategies. Through a CRISPR/Cas9 approach, screens have revealed differences in gene essentiality specific to cell lines and cancer types in addition to overlapping essential genes in leukemia cell lines [8]. Further functional identification has determined essential gene networks and synthetic lethal interactions in acute myeloid leukemia cell lines [9]. Gene essentiality appears to be conditional, and it may depend on mutation strategies, growth conditions, cell lineages, and the compensation of paralogous genes or parallel pathways. Studies in yeast have shown that gene essentiality can be adaptive to various environments and evolvable, probably through whole-chromosome and segmental aneuploidy [10, 11]. The distinction between essential genes and non-essential genes does not appear to be very strict. A quantitative assessment has been proposed to determine gene essentiality [12]. However, in vivo functional insights into gene essentiality remain to be explored in animal models.

Autophagy is an evolutionarily conserved catabolic process, which degrades toxic aggregates and damaged organelles and recycles them as basic building blocks in order to maintain cellular homeostasis [13–15]. Dysregulations of autophagy were associated with neurodegenerative diseases, including retinopathy [16]. Under most pathological conditions affecting the optic nerve, including optic nerve transection, glaucoma, and retinal ischemia, a marked increase in autophagic markers in the RGC has been described [17–21]. However, it remains unclear whether this increase plays a protective or detrimental role under these conditions and whether therapeutic approaches should foster or inhibit autophagy. It seems that maintenance of autophagy homeostasis is important for normal physiological functions of retina.

GCP4 (gamma-tubulin complex protein 4, encoded by *TUBGCP4*) belongs to γ-tubulin ring complexes (γ-TuRCs) [22], which includes GCP4, 5 and 6, and γ-tubulin small complexes (γ-TuSCs: GCP2, GCP3, and γ-Tubulin). *Gfh1* (homolog of human *TUBGCP4*) mutants are viable in fission yeast [23, 24]. Many individuals with the *Dgrip75* (homolog of *TUBGCP4*) mutation are viable; some larvae die after hatching, but both sexes are sterile and have defects in abdominal morphology and the thoracic bristle pattern in *Drosophila* [25, 26]. In an assessment of essentiality for cell survival in the Burkitt’s lymphoma cell line, the CRISPR score has been defined as the average log2-fold-change in the abundance of all sgRNAs targeting a given gene, and genes with a CS < −0.1 and a corrected *p* < 0.05 have been defined as cell essential. On the basis of the cutoff values, 1878 genes have been identified as candidate essential genes, including *TUBGCP4* [8].

In humans, *TUBGCP4* mutations have been identified in patients with autosomal-recessive microcephaly and chorioretinopathy [27]. However, GCP4’s essentiality for embryo survival is unknown. Using knockout mouse models, we determined the gene essentiality of *Tubgcp4* for embryo survival. Haploinsufficiency and dosage compensation of *Tubgcp4* was determined in heterozygous mice. The dosage effect of GCP4 was then assessed in both cell lines and mice. The functions of GCP4 in maintenance of retina homeostasis were determined. We additionally demonstrated GCP4 pathways in regulation of autophagy in the retina.

## Results

### *Tubgcp4* knockout results in early embryonic lethality

To explore the physiological functions of *Tubgcp4* in embryo development, we first generated *Tubgcp4* knockout mice. Gene targeting in ES cells was performed, which generated exon 2–6 deletion and a frameshift after exon 1 (Supplementary Fig. S1a). Two lines of heterozygous mutant mice (*Tubgcp4*^*+/−*^) were generated separately. These *Tubgcp4*^*+/*−^ mice were viable and fertile. There were no differences in growth rate, body fat or lean mass between heterozygous mice and their wild-type littermates (Supplementary Fig. S2), whereas protein levels of GCP4 were not markedly lower in Tubgcp4^*+/*−^ mice than wild type (Supplementary Fig. S3a). Genotype analysis of progeny from heterozygote intercrosses revealed that 36.1% were wild type, 63.9% were heterozygous, and none were homozygous (Table 1 and Supplementary Fig. S1b). This abnormal ratio phenomenon was identical to that in mutant lines derived from two independent ES clones. These results indicated that the *Tubgcp4* homozygous mutation resulted in embryonic lethality.

**Table 1:** Genotyping analysis of the progeny from *Tubgcp4*^*+/−*^ heterozygous intercrosses

Embryo Stage	Total Number	Genotype (Ratio)			N.D.	Resorption
		+ / +	+ /−	−/−		
E3.5	63	16 (1)	34 (2.1)	11 (0.89)	2	0
E4.5	35	9 (1)	17 (1.9)	7 (0.78)	2	0
E5.5	31	8 (1)	18 (2.3)	4 (0.5)	1	0
E6.5	51	14 (1)	26 (1.9)	6 (0.4)	0	5
E7.5	41	10 (1)	24 (2.4)	0 (0)	0	7
Adult	191	69 (1)	122 (1.8)	0 (0)	0	0

To assess the specific period of *Tubgcp4* knockout-induced developmental failure, embryos from heterozygous mating were collected at various periods of gestation, and their genotypes were determined by PCR (Table 1 and Supplementary Fig. S1b). The number of homozygous mutant embryos decreased at E5.5, and no homozygous mutant embryos were detected after E7.5, thus indicating an embryonic death in peri-implantation of *Tubgcp4*-deficient embryos.

**Fig. 1:**
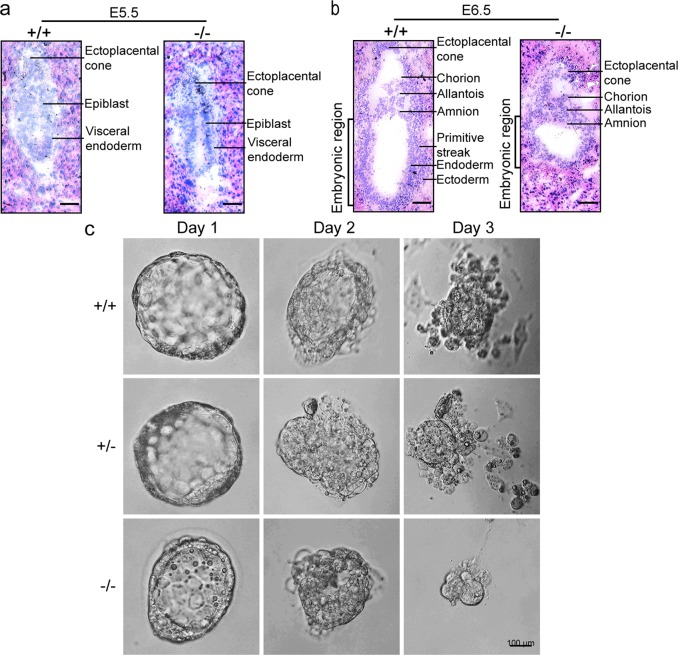
*Tubgcp4* knockout leads to early embryonic lethality. **a**, **b** Histological sections of wild-type and *Tubgcp4*^−/−^ embryos grown in utero. *Tubgcp4*-null and wild-type embryos at E5.5 (**a**) and E6.5 (**b**) were dissected from heterozygous intercrosses. Embryonic tissues were isolated by laser captured microdissection from the sections and were genotyped by nested PCR. Short embryonic region was observed in *Tubgcp4*^−/−^ embryos. Scale bar: 50 µm. **c** Outgrowth of wild-type, heterozygote, and mutant blastocysts in vitro. Blastocysts recovered at E3.5 were cultured in vitro for 3 days and subsequently genotyped by nested PCR. After 3 days of in vitro culture (E3.5+3), outgrowths composed of an ICM on top of a layer of trophoblastic giant cells (TGC) were detected in WT and heterozygous embryos. *Tubgcp4*^*–/–*^ embryos that reach this stage consist either of a very small ICM, remnant TGC or a combination of both. Scale bar: 100 µm

To characterize the structural abnormality of *Tubgcp4*^−/−^ embryos, we sectioned intact deciduas of E5.5 and E6.5 from heterozygous intercrosses. Sectioned embryonic tissues were collected by microdissection for PCR genotyping. All embryos appeared morphologically normal at E5.5, although the proportion of *Tubgcp4*^−/−^ embryos was lower than that in wild type. However, *Tubgcp4*^−/−^ embryos at E6.5 displayed a developmental retardation with a short embryonic region (Fig. 1a, b). To further determine the developmental defects of *Tubgcp4*-deficient embryos, blastocysts were recovered at E3.5 and cultured in vitro for 1–3 days. After culturing for 2 days, abnormal development was observed in *Tubgcp4*-deficient embryos. After day 3, the number of *Tubgcp4*^−/−^ outgrowths decreased, thus indicating that the proliferation of embryonic cells was arrested at E6.5 (Fig. 1c). These results were consistent with the high expression level of GCP4 at E6.5 (Supplementary Fig. S3b).

### GCP4 affects mitotic spindle formation in a dose-dependent manner

**Fig. 2:**
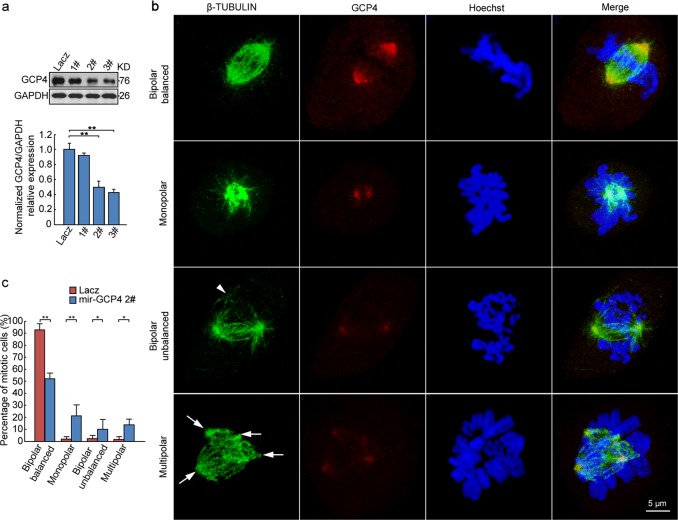
GCP4 knockdown disturbs mitotic spindle formation. **a** GCP4 expression in three mir-GCP4 stably expressing cell lines (1#, 2# and 3#). LacZ miRNA was used as a knockdown control. GAPDH was used as a loading control. Quantification of GCP4 expression is indicated in the bottom panel. GCP4 was efficiently knocked down in both 2# and 3# cell lines. One-way ANOVA followed by Bonferroni posttest was used for statistical analysis. **p < 0.01. **b** Representative images of impaired mitotic spindles. mir-GCP4 stably expressing cell line 2# was used. Endogenous GCP4 and β-TUBULIN were examined by indirect immunofluorescence using anti-GCP4 (red) and anti-β-TUBULIN (green) antibodies. The nuclei were stained with Hoechst reagent. Images were acquired as z-stacks from the top to the bottom of each cell by confocal microscopy. The images are maximum intensity projections from deconvolved z stacks of representative cells. Arrowhead indicates a potential spindle pole; Arrows indicate spindle poles. Bipolar balanced, cells with a broad-based bipolar spindle; Monopolar, cells with only one spindle pole; Bipolar unbalanced, cells with an unequal bipolar spindle; Multipolar, cells with ≥ 3 spindle poles. Scale bar: 5 µm. **c** Quantification of the mitotic spindle types in (**b**). Data were represented as means ± SD (*n* = 3 experiments, with 50 cells/experiment). *p* values were calculated by two-tailed *t-*test: *p ≤ 0.05; **p ≤ 0.01

The developmental retardation of *Tubgcp4*-deficient early embryos suggested a cell division defect. We thus investigated the potential role of GCP4 during mitosis. We attempted to construct a *Tubgcp4* knockout cell line by using CRISP/Cas9 technology but obtained only non-functional mutations (deletions of multiple 3 bases) (Supplementary Fig. S4), thus suggesting an essential role of GCP4 in mitosis. To confirm this possibility, we further constructed *Tubgcp4* knockdown cell lines (Fig. 2a). Immunofluorescence analysis of GCP4 and β-tubulin showed abnormal spindle assembly in these knockdown cell lines, including monopolar spindles, unbalanced bipolar spindles and multipolar spindles (Fig. 2b and Supplementary Fig. S5a). The proportion of normal bipolar balanced spindles was significantly lower in *Tubgcp4* knockdown cell lines than in controls, and the proportion of abnormal spindle types was significantly higher (Fig. 2c and Supplementary Fig. S5b). In particular, the monopolar spindle type increased to 20% (Fig. 2c and Supplementary Fig. S5b). Together, these results suggested a dose-dependent role of GCP4 during mitosis. RNA interference decreased the protein level of GCP4 and affected mitotic spindle formation, whereas complete knockout of GCP4 prevented cell survival and led to embryonic lethality.

### Retinopathy in *Tubgcp4*^+/−^ mice

**Fig. 3:**
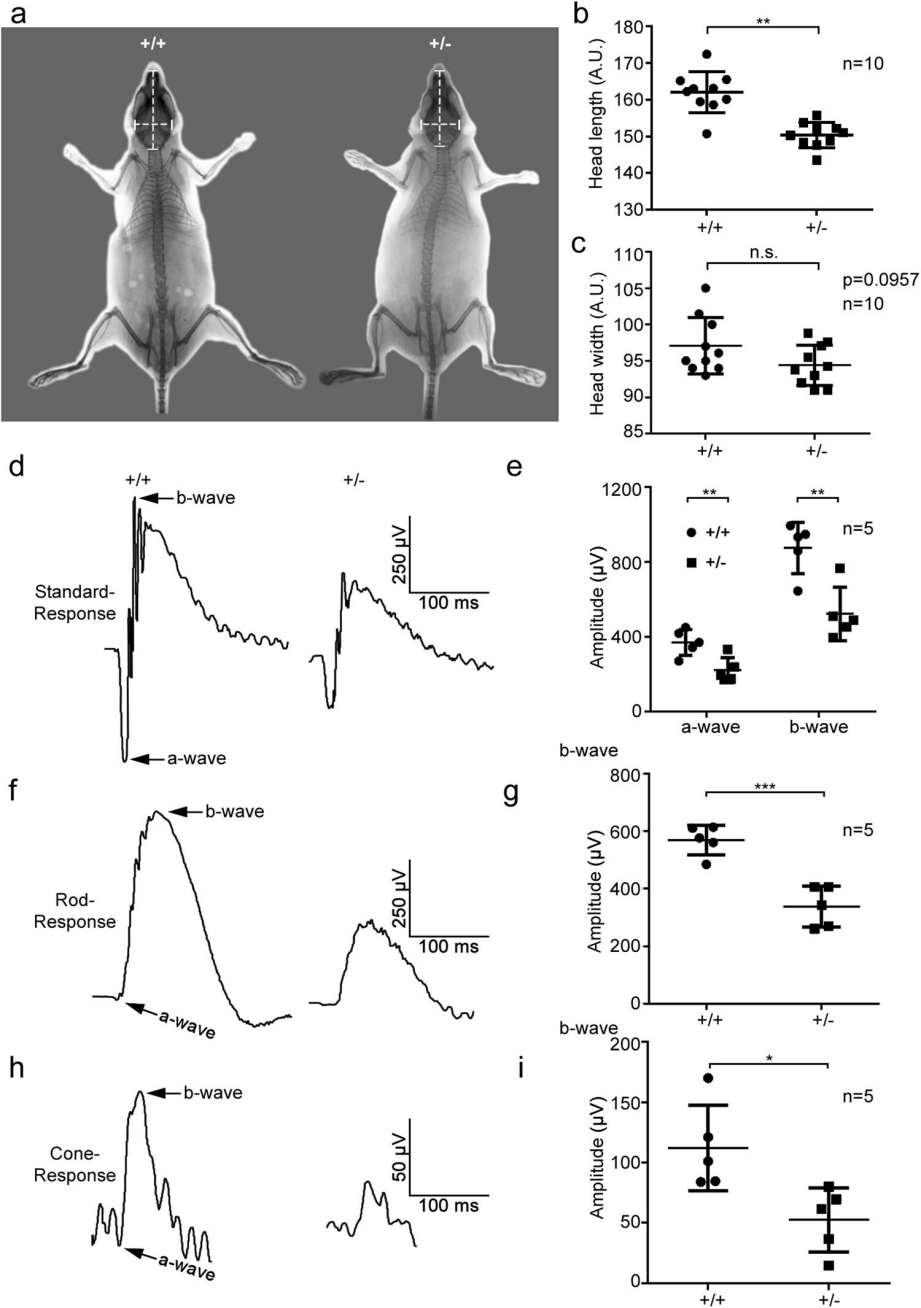
Microcephaly with retinopathy phenotypes in *Tubgcp4*^*+/−*^ mice. **a** X-ray images of *Tubgcp4*^*+/−*^ mice and their wild-type littermates. The biparietal diameter (horizontal dotted line, head width) and the length between distal nasal bone and interparietal bone (vertical dotted line, head length) were measured to analyze size of head. **b**, **c** Statistical analyses of head length (**b**) and head width (**c**). Short head length was observed in *Tubgcp4*^*+/−*^ mice. Data were represented as means ± SD of ten mice. **p < 0.01. **d**, **f**, **h** Saturating ERG responses of the retinas of the mice as indicated to 510 nm flashes at an intensity of −35 log scot. cd. s/m^2^ under scotopic (**d**, **f**) and photopic (**h**) conditions. Each trace is the average of individual records from five mice. **e**, **g**, **i** Statistical analysis of the saturating amplitude of a- and b-waves of the mice as indicated under scotopic (**e**, **g**) and photopic (**i**) conditions. Amplitude levels of a- and b-waves were significantly reduced in *Tubgcp4*^*+/−*^ mice. The *Tubgcp4*^*+/+*^ and *Tubgcp4*^*+/−*^ mice were 6-month-old littermates. Data were represented as means ± SD of five mice. *p-*values were calculated by two-tailed *t-*test, *p *<* 0.05; **p < 0.01

Because GCP4 has a dosage effect on exerting its roles in development, we explored the pathological phenotypes of *Tubgcp4*^+/−^ mice in detail. The head size of heterozygous mice, measured by X-ray imaging of bones, was smaller than that of their wild-type littermates (Fig. 3a–c), an effect mimicking *TUBGCP4* mutant autosomal-recessive microcephaly in humans [27]. We further detected pathological lesions of sensitive tissue retina by using electroretinography (ERG). Under scotopic conditions, standard responses showed that the average saturated a-wave and b-wave decreased ~40% in the heterozygous mice compared with wild-type mice (Fig. 3d, e). These results indicated that the rod- and cone-driven circuits were significantly affected in the retinas of *Tubgcp4*^+/−^ mice (Fig. 3d, e). The rod responses confirmed this result (Fig. 3f, g). Similarly to the scotopic ERG responses, photopic ERG responses were weakened in the b-wave in *Tubgcp4*^+/−^ mice, decreasing approximately 50% compared with that in wild-type mice (Fig. 3h, i). To exclude the possibility that the overserved phenotypes were due to rd8-associated retinal degeneration [28], we conducted a PCR screening for rd8 and found that the mice were free of background mutation of rd8 (Supplementary Fig. S6). These results indicated that haploinsufficiency of *Tubgcp4* led to retinopathy.

### Haploinsufficiency of GCP4 leads to structure disorganization of retina

**Fig. 4:**
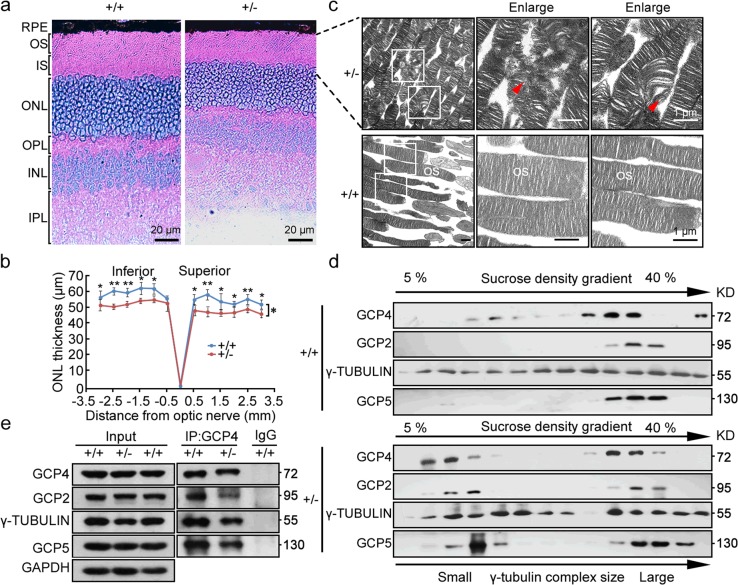
Disorganization of the outer segment (OS) and disassembly of γ-TuRC in retinas of *Tubgcp4*^*+/−*^ mice. **a** Histologic analysis of wild-type and heterozygous retinas at age of 6 month. RPE, retinal pigment epithelium; OS, outer segment; IS, inner segment; ONL, outer nuclear layer; OPL, outer plexiform layer; INL, inner nuclear layer; IPL, inner plexiform layer; GCL, ganglion cell layer. Scale bar: 20 µm. **b** Statistical analysis of ONL thickness in *Tubgcp4*^*+/+*^ and *Tubgcp4*^*+/−*^ mice (*n* = 3). The ONL thickness was measured along the vertical meridian at each defined distance from the optic nerve head. Data were represented as mean ± SD. Two-way ANOVA followed by Bonferroni posttest was used for statistical analysis. *p *<* 0.05; **p < 0.01. **c** Transmission electron microscopy of heterozygous (top) and wild-type (bottom) photoreceptors showed a disorganized OS in the heterozygous retinas. Ultrathin sections were made to show the longitudinal axis of the photoreceptor cell. Red arrowheads indicated degenerated OS discs in the heterozygous retinas; Scale bar:1 µm. **d** Immunoblots of extracts from wild-type and heterozygous retinas after fractionation in gradients of 5–40% sucrose. Antibodies against γ-TUBULIN and GCP2, GCP4, and GCP5 were used for visualizing γ-TuRC components. **e** Co-immunoprecipitation analysis showed that GCP4 interaction with GCP2, GCP5, and γ-TUBULIN was impaired in retinas of *Tubgcp4*^*+/−*^ mice in comparison with wildtype. Retina cell lysates were extracted for immunoprecipitation with anti-GCP4 (Mouse IgG for control) followed by immunoblotting with antibody against γ-TUBULIN and GCP2, GCP4, and GCP5 for visualizing γ-TuRC components. GAPDH was used as an internal control

We then assessed retina morphology in *Tubgcp4*^+/−^ and age-matched *Tubgcp4*^*+/+*^ mice. Histologic analysis of heterozygous retinas at the age of 6 months revealed a decrease (10–20%) in the thickness of the outer nuclear layer (ONL) in heterozygous mice compared with wild-type littermates (Fig. 4a, b). Ultrastructural analysis of photoreceptors in mutant retinas showed a disorganized outer segment morphology and disrupted lamellar structure of the outer segment (OS) (Fig. 4c).

Because GCP4 localizes with γ-tubulin as γ-TuRC in centrosomes [22], we examined whether GCP4 depletion might affect γ-TuRC formation in *Tubgcp4*^*+/−*^ retinas. Extracts from wild-type and heterozygous retinas were subjected to sucrose gradient sedimentation and subsequent western blot analysis to detect different γ-TuRC components. In wild-type, GCP4 was detected mainly in higher molecular weight fractions, together with GCP2 and γ-tubulin (Fig. 4d, top). However, in *Tubgcp4*^*+/−*^ retinas, GCP4 protein shifted to lower molecular weight fractions, thus indicating the disassembly of γ-TuRC (Fig. 4d, bottom). Further co-immunoprecipitation analysis confirmed the disassembly of γ-TuRC in retinas of *Tubgcp4*^*+/−*^ mice (Fig. 4e**)**. These results suggested that the haploinsufficiency of GCP4 affected the assembly of γ-TuRC and led to photoreceptor degeneration in retina.

### Upregulation of autophagy in *Tubgcp4*^+/−^ retinas

**Fig. 5:**
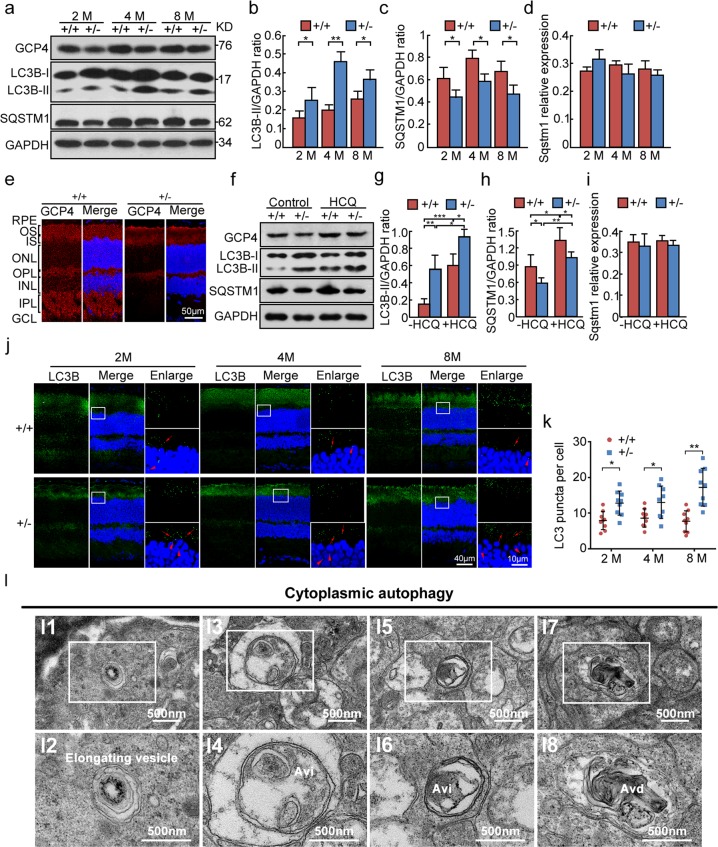
Upregulation of autophagy in the heterozygous retinas. **a** Western blot analysis showed that LC3B-II was upregulated and SQSTM1 was downregulated in the heterozygous retinas in comparison with the wild-type retinas. GAPDH was used as an internal control. **b**, **c** Quantification of LC3B-II and SQSTM1 expression levels in retinas in (**a**). The results were representative of 3 independent experiments and represented as means ± SD. The data were analyzed using Student’s *t*-test. *p < 0.05; **p < 0.01. **d** Real-time quantitative PCR of *Sqstm1* in these retinas. *β-actin* was used as an internal control. **e** Immunofluorescence analysis of GCP4 in wild-type and heterozygous retinas using anti-GCP4 antibody. GCP4 was mainly expressed in OS, IS, OPL, IPL, and GCL of retina in both wild-type and heterozygous mice. The nuclei were stained with Hoechst reagent. RPE, retinal pigment epithelium; OS, outer segment; IS, inner segment; ONL, outer nuclear layer; OPL, outer plexiform layer; INL, inner nuclear layer; IPL, inner plexiform layer; GCL, ganglion cell layer. Scale bar: 50 μm. **f**–**i** HCQ treatment resulted in accumulation of both LC3-II and SQSTM1 proteins in both wild-type and heterozygous retinas. The retina lysates were analyzed by immunoblotting with antibodies as indicated. GAPDH was used as an internal control. **g**, **h** Quantification of LC3B-II and SQSTM1 protein levels in retinas in (**f**). The results were representative of 3 independent experiments and represented as means ± SD. The data were analyzed using one-way ANOVA followed by Bonferroni posttest. *p < 0.05; **p < 0.01; ***p < 0.001. **i** Real-time quantitative PCR of *Sqstm1* in retinas in (**f**). *β-actin* was used as an internal control. The data were analyzed using one-way ANOVA followed by Bonferroni posttest. **j** Immunofluorescence analysis of the LC3B puncta in the heterozygous and wild-type retinas of 2, 4, and 8 months using anti-LC3B antibody. LC3B puncta were mainly located in the cytoplasm of photoreceptor cells in both heterozygous and wild-type retinas (red arrows). Some LC3B puncta were also observed in the nuclei of ONL in heterozygous retinas (red arrowheads). The nuclei were stained with Hoechst reagent. The enlarged images were originated from the white squares. **k** Statistic analysis of LC3 puncta per cell using Student’s *t*-test. Three retinas from three mice were sectioned at indicated age and three sections were counted per retina. Data were represented as means ± SD. *p < 0.05; **p < 0.01. **l** Transmission electron microscopy of autophagosomes in photoreceptor cells. Representative images of autophagosomes in the cytoplasm of photoreceptor cells. The images in the white squares in panels **l1**, **l3**, **l5**, and **l7** were enlarged and showed in panels **l2**, **l4**, **l6**, and **l8**, respectively. Avi, initial autophagic vacuole; Avd, degradative autophagic vacuole. Scale bars are indicated in each image

To further investigate molecular mechanisms of photoreceptor degeneration in the retina, we detected autophagy in retinas of mice at different ages. The GCP4 protein level was slightly lower in heterozygous retinas than wild-type retinas, particularly at 4 months (Fig. 5a–e). Western blot analysis showed that LC3B-II, a key autophagy protein, was upregulated and its downstream substrate SQSTM1 was downregulated in the heterozygous retinas in comparison with the wild-type retinas (Fig. 5a–c). Given that *Sqstm1* expression is regulated at transcriptional level when prolong starvation treatment [29], we performed qPCR assay to assess *Sqstm1* transcription and found that there was no significant change between these groups (Fig. 5d). Further, the retinal cells were used to assess GCP4-involved autophagic flux. Hydroxychloroquine (HCQ, an autophagy flux inhibitor in vivo [30]) treatment resulted in accumulation of both LC3-II and SQSTM1 proteins in both wild-type and heterozygous retinas (Fig. 5f–i). In addition, over-expression of GCP4 inhibited autophagy in the 293T cell line (Supplementary Fig. S7). Thus, the decrease in GCP4 protein levels was associated with the upregulation of autophagy in the heterozygous retinas. Furthermore, in *Tubgcp4*^*+/−*^ retinas, LC3B puncta were detected at photoreceptor inner segments and near the nuclei, thus indicating autophagosome formation in the segment (Fig. 5j). The numbers of LC3B puncta were significantly higher in *Tubgcp4*^*+/−*^ retinas than wild-type retinas (Fig. 5k). Transmission electron microscopy of *Tubgcp4*^*+/−*^ retinas confirmed the formation of autophagosomes in the photoreceptor inner segment (Fig. 5l). In addition, nuclear autophagy could probably occur in photoreceptor cell segments (Supplementary Fig. S8). To address whether depletion of GCP4 affects phagocytic ability in heterozygous retinas [31], we performed an immunofluorescent examination on both retinal sections and retinal pigment epithelium (RPE) flat mounts, and observed no significant change in the phagocytic ability of RPE between heterozygous and wild-type retinas (Supplementary Fig. S9). These results suggested that autophagy was upregulated in the *Tubgcp4*^*+/−*^ mouse retinas and GCP4 was involved in autophagy regulation in retina.

### Autophagy inhibition reduces retinal degeneration in *Tubgcp4*^+/−^ retina

**Fig. 6:**
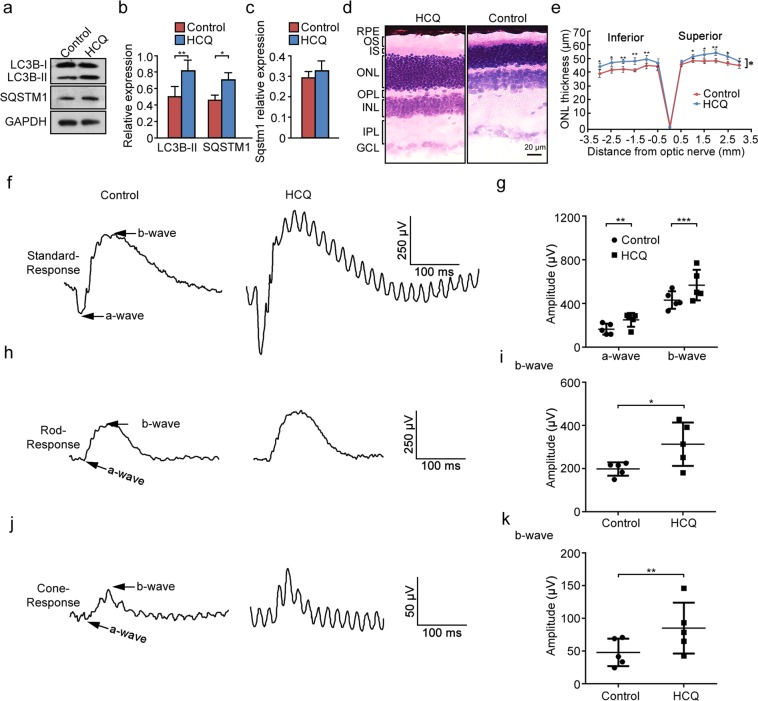
Autophagy inhibition by HCQ treatment in vivo increased photoreceptor survival and rescued retinal function in GCP4 heterozygotes. **a** Western blot analysis showed that LC3B-II and SQSTM1 was accumulated in heterozygous retinas after 2 months of HCQ treatment via the drinking water. GAPDH was used as an internal control. **b** Quantification of LC3B-II and SQSTM1 expression levels in retinas in panel **a**. **c** Real-time quantitative PCR of *Sqstm1* in retinas in (**a**). *β-actin* was used as an internal control. The results were representative of 3 independent experiments and represented as means ± SD. The data were analyzed using Student’s *t*-test. *p < 0.05; **p < 0.01. **d** Histologic analysis of heterozygous retinas with or without HCQ treatment at age of 8 month. RPE, retinal pigment epithelium; OS, outer segment; IS, inner segment; ONL, outer nuclear layer; OPL, outer plexiform layer; INL, inner nuclear layer; IPL, inner plexiform layer; GCL, ganglion cell layer. Scale bar: 20 µm. **e** Statistical analysis of ONL thickness in *Tubgcp4*^*+/−*^ mice with or without HCQ treatment (*n* = 3). The ONL thickness was measured along the vertical meridian at each defined distance from the optic nerve head. Data were represented as mean ± SD. Two-way ANOVA followed by Bonferroni posttest was used for statistical analysis. *p *<* 0.05; **p < 0.01. **f**, **h**, **j** Saturating ERG responses of the retinas of the mice as indicated to 510 nm flashes at an intensity of −35 log scot. cd. s/m^2^ under scotopic (**f**, **h**) and photopic (**j**) conditions. Each trace is the average of individual records from five mice. **g**, **i**, **k** Statistical analysis of the saturating amplitude of a- and b-waves of the mice as indicated under scotopic (**g**, **i**) and photopic (**k**) conditions. Amplitude levels of a- and b-waves were significantly rescued in the HCQ-treated *Tubgcp4*^*+/−*^ mice. The mice were 8-month-old littermates. Data were represented as means ± SD of five mice. *p-*values were calculated by two-tailed *t-*test, *p *<* 0.05; **p < 0.01

Given the photoreceptor degeneration when autophagy was increased, we test whether autophagy inhibition can rescue the retinopathy phenotypes in heterozygous mice. To decrease autophagy activity in the retinas of *Tubgcp4*^+/−^ mice, we administered mice with HCQ via the drinking water for 2 months. HCQ was effective in reducing the flux as evidenced by accumulation of LC3-II and SQSTM1, but there was no significant change at transcription levels of *Sqstm1* (Fig. 6a–c). HCQ treatment resulted in an obvious increase of ONL thickness in both the superior and inferior retina (approximately 40% after 2 months of HCQ-treatment in comparison with controls) (Fig. 6d, e). Moreover, ERG showed a significant increase in retinal function in HCQ-treatment mice (Fig. 6f–k). These findings suggested that decrease of autophagy activity in the heterozygous retinas increased photoreceptor survival and rescued retinal function.

### GCP4 inhibits autophagy by competing with ATG3 for interaction with ATG7

**Fig. 7:**
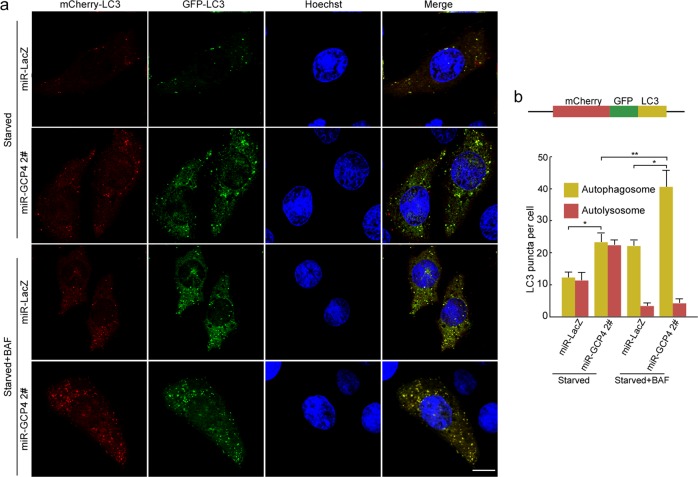
Autophagy flux associated with GCP4. **a** Detection of autophagy flux using fluorescent confocal microscopy. Stable miR-*Gcp4*-2# and miR-*LacZ* COS-7 cells were transfected with a tandem expression vector mCherry-GFP-LC3 and cultured in normal and EBSS medium for 2 h. In the BAF + EBSS group, the cells were treated with BAF for 4 h to suppress the fusion between autophagosome and lysosome. Yellow or green puncta indicated autophagosomes, while red puncta include autophagosomes and autolysosomes, because GFP protein is sensitive and attenuated in an acidic environment of autolysosome. Scale bar: 10 μm. **b** The tandem structure of mCherry-GFP-LC3 and statistical analysis of LC3 puncta per cell (*n* *=* 3 experiments, with 40 cells/experiment). Data were represented as means ± SD. Two-way ANOVA followed by Bonferroni posttest was used for statistical analysis. *p < 0.05; **p < 0.01

To explore the molecular mechanisms of GCP4 in the regulation of autophagy, we analyzed autophagy flux through a tandem fluorescent indicator, mCherry-GFP-LC3. Because green fluorescence of the fusion protein is very sensitive to the acidic environment of lysosomes and is quickly quenched in autolysosomes, only red fluorescence could be detected in the autolysosomes [32, 33]. Confocal microscopy analysis using the indicator system in COS-7 cells showed that GCP4 knockdown promoted the formation of autophagosomes after starvation induction. With BAF treatment, autophagosomes clearly accumulated, and the number of autolysosomes decreased (Fig. 7a, b and Supplementary Fig. S10). These results suggested that GCP4 inhibits autophagosome formation.

**Fig. 8:**
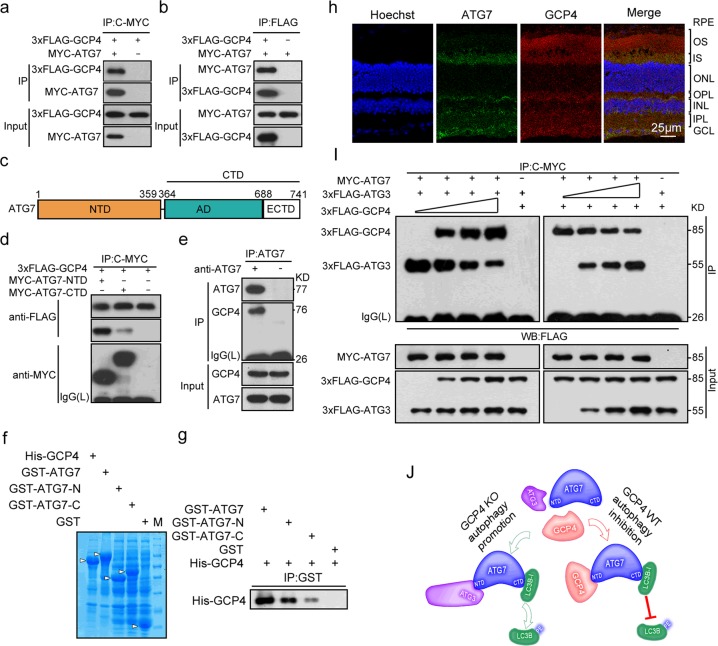
GCP4 inhibits autophagy by competing with ATG3 to interact with ATG7. **a**, **b** Co-immunoprecipitation analysis showed that GCP4 interacted with ATG7 in HEK293T cells. The 293T cells were transiently transfected with both pMyc-*ATG7* and p3xFlag-*TUBGCP4*, and after transfection for 48 h, the whole cell lysates were extracted for co-immunoprecipitation with anti-MYC (**a**) or anti-FLAG (**b**). Anti-FLAG (**a**) or anti-MYC (**b**) antibody was used for Western blotting. **c** Schematic diagram of the ATG7 domains. NTD, N-terminal domain; CTD, C-terminal domain; AD, adenylation domain; ECTD, extreme C-terminal domain. **d** Co-immunoprecipitation analysis showed that GCP4 interacted with the NTD of ATG7. p3xFlag-*TUBGCP4* was co-transfected with pMyc-*ATG7*-NTD, pMyc-*ATG7*-CTD into 293T cells. For co-immunoprecipitation, the lysates were immunoprecipitated with the anti-MYC antibody, followed by immunoblotting with the anti-FLAG antibody. The result showed that FLAG-GCP4 can interact with MYC-ATG7-NTD. **e** GCP4 can interact with ATG7 in vivo. Retina cell lysates were extracted for immunoprecipitation with anti-ATG7 (Rabbit IgG for control) followed by immunoblotting with antibody against GCP4. **f**, **g** GST-pulldown assay showed GCP4 interaction with the N-terminal of ATG7. Coomassie gel showed expression of His-GCP4, GST-ATG7, GST-ATG7-N, GST-ATG7-C, and GST in supernatant of *E. coli* culture (**f**). His-tagged GCP4 was incubated with GST-ATG7, GST-ATG7-N, GST-ATG7-C and GST respectively. Proteins pulled down with glutathione-agarose were subjected to SDS-PAGE followed by immunoblotting with anti-GCP4 antibody (**g**). **h** Co-localization of ATG7 and GCP4 in OS, IS, OPL, IPL, and GCL of retinas by immunofluorescence analysis using anti-GCP4 (second antibody, Alexa Fluor 594-conjugated-goat anti-Rabbit) and Alex488-labeled anti-ATG7. The nuclei were stained with Hoechst reagent. RPE, retinal pigment epithelium; OS, outer segment; IS, inner segment; ONL, outer nuclear layer; OPL, outer plexiform layer; INL, inner nuclear layer; IPL, inner plexiform layer; GCL, ganglion cell layer. Scale bar: 50 μm. **i** GCP4 competes with ATG3 to interact with ATG7. pMyc-*ATG7* was co-transfected with p3xFlag-*ATG3* (1 μg) or p3xFlag-*TUBGCP4* (1 μg) and an increasing amount (0, 0.3, 0.6, and 1.2 μg) of p3xFlag-*TUBGCP4* (left panel) or p3xFlag-*ATG3* (right panel). The cell lysates were immunoprecipitated with the anti-MYC antibody, followed by immunoblotting with the anti-FLAG antibody. The whole cell lysates were examined by western blotting using the anti-FLAG or anti-MYC antibody (input). **j** A model of GCP4 inhibiting autophagy through competing with ATG3 to interact with ATG7. The free ATG7, which is a key autophagy protein to initiate the formation of phagophores, can interact with either ATG3 or GCP4 through its NTD. When *Tubgcp4* is knocked out, ATG3 interacts with ATG7 to promote lipidation of LC3B and autophagy. In wild-type cells, GCP4 can compete ATG3 to interact with ATG7, which inhibits lipidation of LC3B and autophagy. Thus, GCP4 balances functions of ATG3 in autophagy regulation

To further investigate how GCP4 regulates autophagy, GCP4 interacting proteins involved in autophagy initiation were identified through co-immunoprecipitation and co-localization analysis. We determined that ATG7, a key protein for autophagy initiation, can interact with GCP4 in 293T cells (Fig. 8a, b). Truncation mutation analysis showed that GCP4 bound to the N-terminal domain of ATG7 but not to the C-terminal region (Fig. 8c, d).

We further confirmed the interaction in vivo. Co-immunoprecipitation analysis showed that GCP4 interacted with ATG7 in mouse retinas (Fig. 8e). GST-pulldown assays were used to test whether the interaction was direct or indirect. The direct association was observed between GCP4 and ATG7, particularly its N-terminal (Fig. 8f, g). Immunofluorescence analysis of the retinal sections showed that both GCP4 and ATG7 proteins co-localized in the outer segment, inner segment, outer plexiform layer, inner plexiform layer and ganglion cell layer of retina (Fig. 8h). Because ATG3 interacts with the N-terminal domain of ATG7 and consequently promotes autophagy, we further determined the interaction modes of GCP4, ATG3, and ATG7. Co-immunoprecipitation analysis showed that GCP4 competed with ATG3 for interaction with ATG7 (Fig. 8i, j). Together, these results suggested that GCP4 inhibits autophagy by competing with ATG3 to interact with ATG7.

## Discussion

The retina consists of highly specialized and multilayered neural tissue for initial capture and processing of visual signals. Maintenance of retinal homeostasis is critical for normal physiological function of retina, which is often affected by a variety of both physiological and pathological conditions, including genotoxicity, age-associated alterations, light damage, abnormal apoptosis and autophagy [16, 34]. Using knockout mouse models, we have found that GCP4 depletion affected the assembly of γ-TuRC, which led to abnormal spindle formation, thus structure disorganization of the retina. On the other hand, GCP4 can down-regulate autophagy by competing with ATG3 for interaction with ATG7 and interferes with the lipidation of LC3B. In *Tubgcp4* heterozygous retina, haploinsufficiency of GCP4 releases more ATG7 proteins for interaction with ATG3 to up-regulate autophagy. HCQ treatment in vivo to decrease autophagy activity in the heterozygous retinas can increase photoreceptor survival and rescue retinal function. Thus, GCP4 may play bi-functional roles in maintenance of retinal homeostasis, through participating in assembly of γ-TuRC, on the other side, in regulating autophagy in retina. In line with these results, inhibition of autophagy reduced retinal degeneration by pharmacological treatment and *Atg5* knockout in mice [30].

Assessment of gene essentiality in vivo has posed technical and conceptual challenges in mammals. Here, we demonstrated that *Tubgcp4* is an essential gene for early embryo survival in mice. Gene essentiality was assessed in terms of the dosage effect of *Tubgcp4* on mitosis and retina homeostasis. GCP4 regulates autophagy through the ATG3/GCP4-ATG7-LC3B pathway, which plays key roles in the maintenance of retina architecture in a dose-dependent manner. Accordingly, we propose that the essential gene *Tubgcp4* has dose-dependent effects from tissue homeostasis to early embryo survival.

An intriguing finding in this study is the dosage effect of essential genes. In mammals, two copies of gene products are essential for embryo survival. In heterozygotes, embryo survival can be rescued by the wild-type allele through dosage compensation. When an essential gene is lacking, the early embryo can still survive until implantation because of the compensation by maternally inherited gene products. In addition, a mammalian pre-implantation embryo has a certain degree of plasticity and an ability to efficiently adapt to its development before implantation [35]. However, haploid dosage is insufficient for the functions of sensitive tissues, such as in the maintenance of retinal homeostasis. The viability of yeast cells with disruptions in components of essential pathways was rescued by aneuploidy of specific chromosomes [11], thereby supporting the dosage compensation effect of essential genes observed in mammals. Thus, gene essentiality should be assessed as a dosage effect.

Molecular and cellular mechanisms underlying embryo lethality are not well understood, although several scenarios have been proposed, such as impaired expansion of the primitive streak with Oct4 depletion [36], impaired placental development caused by insufficient oxygen and nutrient transfer in *Pdcd5*-deficient embryos [37], and lesions in inner cell mass proliferation in mTOR knockout embryos [38]. Here, we propose that GCP4 is essential for mitotic spindle assembly in a dose-dependent manner and that depletion of GCP4 leads to abnormal spindle assembly and thus embryonic lethality. GCP4, together with other subunits of γ-TuRC, participate in the nucleation of spindle microtubules [39, 40]. In fission yeast cells, *Gfh1* (homolog of *TUBGCP4*) mutants are viable, but with quantitatively less nucleation [23, 24]. In *Drosophila*, most of *Dgrip75* (homolog of *TUBGCP4*) mutants are viable, but both males and females are sterile [25, 41]. *TUBGCP4* siRNA affects spindle orientation in HeLa cells [42, 43]. Our study demonstrated that *Tubgcp4* knockout in mice resulted in embryonic lethality; concordantly, no homozygous mutants have been observed in human families with autosomal-recessive microcephaly and chorioretinopathy [27]. These data suggest that the gene essentiality of *Tubgcp4* in embryonic survival is specific to mammals. However, in fission yeast and *Drosophila*, *Gfh1/Dgrip75* mutants can survive even though microtubule nucleation is impaired in culture cells, thus suggesting that its function can be compensated by other microtubule-associated pathways. Thus, our study reveals a novel genetic cause of embryonic lethality, partially explains the etiology of infertility, and suggests that essential genes can be used as candidate markers in genetic counseling.

The light-sensitive tissue, retina, links the optic nerve to the brain. Various environmental insults often impair retinal function and lead to retinal diseases. Autophagy is an essential mechanism for cellular renovation in maintaining retinal homeostasis [16, 44]. Under most pathological conditions affecting the optic nerves, including optic nerve transection, glaucoma, and retinal ischemia, an upregulation of autophagy in the retina has been detected [17–21]. Autophagy deficiency due to depletion of *Beclin1* or *Atg7* results in light-induced retinal degeneration [45]. Thus, homeostasis of autophagic activity is essential to supporting retinal function. Nevertheless, regulation mechanisms of autophagy in maintaining retinal homeostasis remain elusive. Here, we have identified a pathway of ATG3/GCP4-ATG7-LC3B in the regulation of autophagy in retina, which is schematically shown in Fig. 8j. GCP4 inhibits autophagy by competing with ATG3 for interaction with ATG7 and interferes with the lipidation of LC3B. The autophagic regulation pathway has both physiological and pathologic implications in understanding of the molecular mechanisms underlying retina degeneration. Our results also have clinical significance in the potential treatment of retinopathy through the dosage effect of essential gene GCP4.

## Materials and methods

### Animals

Wild-type C57BL/6J and CD-1 mice were purchased from Shanghai Biomodel Organism Science & Technology Development (Shanghai, China). All animal experiments and methods were performed in accordance with the relevant approved guidelines and regulations, as well as under the approval of the Ethics Committee of Wuhan University.

### Generation of *Tubgcp4* knockout mice and genotyping

A 5.2-kb DNA fragment containing a region of exon 2 to exon 6 of *Tubgcp4* was replaced with a 1.9-kb Kanamycin-Neomycin cassette and was used as a dominant selection marker (Supplementary Fig. S1a). The herpes simplex virus-thymidine kinase (*HSV-tk*) was used as a negative selection marker. Gene targeting was performed in SCR012 ES cells (129/S6/SvEv). ES cell culture and electroporation were performed as previously described [46]. The targeted ES clones were verified by PCR (Table S1). Two independent targeted ES clones were injected into C57BL/6 J blastocysts, which were transferred into a CD-1 foster mother. The resulting male chimeras were mated with C57BL/6J females to establish knockout mouse lines. Both lines exhibited identical phenotypes.

Genotyping was carried out with the nested PCR method. Genotyping of adult and post-implantation embryos was performed by multiplex PCR using a mixture of three primers: GT-f1 + GT-r and GT-f2 + GT-r (Table S1), whereby the wild-type alleles yielded a band of 421 bp and the knockout allele produced a band of 585 bp. Genotyping of pre-implantation embryos was performed using a nested PCR strategy, whereby 1 µl from the first round of PCR amplification was used as a template for the second round of PCR amplification using primers GT-nest-f1 + GT-nest-r and GT-nest-f2 + GT-nest-r (Table S1). The wild-type *Tubgcp4* allele yielded a 360-bp product and the knockout alleles yielded a 485-bp product (Supplementary Fig. S1b).

For genotyping of in situ embryos in utero, embryo tissues were cryosectioned (Leica, Wetzlar, Germany). Some sections were used for H.E. staining, and others were dissected by laser captured microdissector (LMD6500, Leica). Then, embryo samples were collected in PCR tubes and were lysed in 10 μL lysis buffer (0.005% SDS + 1 mg/mL protein kinase K) for 1 h at 55 °C and then 10 min at 98 °C. Approximately 1 μL lysate was used as a template for genotyping as described above for pre-implantation embryos.

### Antibodies

The primary antibodies were as follows: anti-GCP4 (D-5) (sc-271876, Santa Cruz Biotechnology, Dallas, USA) for western blotting, immunofluorescence and co-immunoprecipitation (co-IP) analysis; Alexa Fluor488-ATG7 (ab214867, Abcam) and anti-GCP4 (GTX115949, Gene Tex, Irvine, USA) for immunofluorescence analysis of histological section of the retina; and anti-GCP5 (14620-1-AP, Proteintech Group, Rosemont, USA), anti-γ-tubulin (15176-1-AP, Proteintech Group), anti-GCP2 (AP12746C, Abgent, San Diego, USA), anti-ATG7 (ab133528, Abcam, Cambridge, USA) for western blotting and co-IP; anti-LC3B (3868 s, Cell Signaling Technology, Danvers, USA) and anti-TUBB3 (AC008, ABclonal Biotechnology, Wuhan, China)  for immunofluorescence and Western blotting; anti-p62 (SQSTM1) (18420-1-AP, Proteintech Group), anti-GAPDH (CW0100, CWBIO, Beijing, China), anti-FLAG (F3165, Sigma-Aldrich, St Louis, USA), and anti-MYC/c-MYC (11667149001, Roche Applied Science, Indianapolis, USA) for Western blotting and co-immunoprecipitation.

The following secondary antibodies were used: peroxidase-conjugated AffiniPure goat anti-mouse IgG, light chain specific (115-035-174, Jackson ImmunoResearch Laboratories, West Grove, USA), horseradish peroxidase (HRP) conjugated-goat anti-mouse IgG (H + L) secondary antibody (31430, Invitrogen, Carlsbad, USA), and HRP conjugated-goat anti-Rabbit IgG (H + L) secondary antibody (31460, Invitrogen).

The following fluorescent antibodies were used: Alexa Fluor 594-conjugated-goat anti-Rabbit IgG (H + L) secondary antibody (R37117, Invitrogen), Alexa Fluor 488-conjugated-goat anti-Rabbit IgG (H + L) secondary antibody (R37116, Invitrogen), and TRITC conjugated-goat anti-Mouse IgG (H + L) secondary antibody (A16071, Invitrogen).

### Plasmid constructs

Mouse *Tubgcp4* CDS (NM_153387.3) was cloned into pCMV-3xFlag using E*coR*I and X*ho*I to generate FLAG-Tubgcp4. Human *ATG7* CDS (NM_006395.2) was cloned into pEGFP-N1 (GM-1013P031, Clontech, Mountain View, USA) and pcDNA3.0-myc using E*coR*I and S*al*I to generate pMyc-ATG7. *ATG7* fragments consisting of MYC-ATG7 residues 1 to 359 or 360 to 741 were amplified using the PCR primers described in Table S1. These fragments were digested with E*coR*I and S*al*I and ligated into pcDNA3.0-myc to generate pMyc-ATG7-NTD and pMyc-ATG7-CTD, respectively. Human *ATG3* CDS (NM_022488.4) was cloned into pCMV-3xFlag using E*coR*I and X*ho*I to generate FLAG-ATG3. LentiCRISPRv2-GCP4-gRNA was constructed as described previously [47]. Briefly, GCP4-gRNAs were designed according to the CRISPR Design Tool (http://crispr.mit.edu/) and synthesized with a B*smB* I sticky end, then annealed and inserted into the lentiCRISPRv2 plasmid (52961, Addgene, Cambridge, USA), which had been digested with B*smb* I (Fermentas, Vilnius, Lithuania). The target sequences are described in Table S1. pcDNA6.2-GW/EmGFP-miR (Invitrogen) was digested by D*ra*I (Fermentas) to remove GFP to generate pcDNA6.2-GW-miR plasmid. To generate the miR-*Tubgcp4* plasmid, *Tubgcp4* specific miRNA and control miRNA target sequences were synthesized and cloned into pcDNA6.2-GW-miR. The target sequences for *Tubgcp4* and *LacZ* are described in Table S1. The plasmid mCherry-GFP-LC3 was a kind gift from Dr. Mingzhou Chen.

### Cell culture, treatment, and transfection

HEK293T, COS-7, and MEF cells were cultured in DMEM (SH30022.01B, HyClone, Logan, USA) with 10% FBS (P30-330250, PAN-Biotech, Aidenbach, Germany). Cells were transfected in 12/24-well plates by using Lipofectamine 2000 (11668027, Invitrogen) according to the routine protocol. To establish stable *Tubgcp4* knockdown cell lines, COS-7 cells were transfected with miR-*Tubgcp4* 1#, miR- *Tubgcp4* 2# and miR- *Tubgcp4* 3# plasmids by using Lipofectamine 2000. Stably expressing cells were screened with blasticidin (15205, Sigma-Aldrich) at a final concentration of 20 µg/mL for 2 weeks. For starvation treatment, the cells were cultured in EBSS medium (SH30029.02, HyClone). For BAF treatment, Bafilomycin A1 (B1793, Sigma-Aldrich) was added to the culture for 4 h before harvesting.

### Western blot analysis and co-immunoprecipitation assays

Western blot analysis was performed as described previously [48]. Briefly, protein extracts (50 μg) from tissues and cell lines were separated in 12% SDS-polyacrylamide gels and then transferred onto 0.45-µm membranes (Amersham Pharmacia Biotech, Hybond-P). Primary antibodies were incubated with the membranes overnight at 4 °C. The membranes were washed in TBST (20 mM Tris-HCl pH 7.5, 150 mM NaCl, 0.1% Tween 20) 3–5 times, incubated with the indicated HRP-conjugated secondary antibody for 1 h at room temperature and then washed in TBST 3–5 times. A Super Signal Chemiluminescent Substrate system (K-12045-D50, Advansta, Menlo Park, USA) was used to detect the signals.

Co-immunoprecipitation was performed as described previously [49]. Briefly, 293T cells were co-transfected with related plasmids by using Lipofectamine 2000. The cells or tissues were lysed in HEPES buffer (50 mM HEPES at pH 7.5, 150 mM NaCl, 1 mM MgCl_2_, 1 mM EGTA, and 0.5% NP-40 with protease inhibitor cocktail (04693159001, Roche Applied Science)). Cell lysates were incubated with the specified antibodies and Protein G agarose (Roche) overnight at 4 °C. The resins were collected by centrifugation and then washed four times with HEPES buffer. Bound proteins were eluted with loading buffer (50 mM Tris-HCl, 2% SDS, 1% mercaptoethanol, 10% glycerol, 0.1% bromophenol blue, pH 6.8), separated by SDS-PAGE and immunoblotted with appropriate antibodies.

### GST pull-down assays

GST pull-down experiments were performed as described previously [50]. Briefly, GST, GST-ATG7, GST-ATG7-N, GST-ATG7-C or His-GCP4 were introduced into *E.coli* BL21 (DE3) pLyS, and the fusion proteins were induced with 0.1 mM IPTG at 16 °C for 24 h. 10 μg supernatants of GST-fusion proteins were mixed with glutathione-agarose beads (Cat# P2020, Solarbio, Beijing, China) for 4 h at 4 °C. The beads were washed three times with HEPES buffer. Then the beads were incubated with 10 μg supernatants of His-GCP4 and rotated at 4 °C overnight respectively. Finally, the glutathione-agarose beads were washed four times with HEPES buffer, and then subjected to Western blot analysis.

### Immunofluorescence analysis

Immunofluorescence analysis was performed as described previously [51]. Briefly, retina tissues were embedded in OCT medium (4583, Tissue-Tek, Miles, USA) and cut into a series of 8-µm sections using a cryostat (Leica). The sections were fixed with pre-cooled methanol for 20 min at −20 °C and permeabilized with 0.5% Triton X-100 (9002–93–1, Sigma-Aldrich) in PBS for 10 min. Alternatively, COS-7 cells on coverslips were extracted with microtubule stabilization buffer (80 mM Pipes, pH 6.8, 1 mM MgCl_2_, 5 mM EGTA and 0.5% Triton X-100) and then fixed with 4% paraformaldehyde for 20 min at room temperature. Both tissue sections and cells on coverslips were treated with 2% BSA for 20 min at room temperature and incubated with indicated primary antibody overnight at 4 °C. After being washed 3 times with PBS, the samples were subjected to appropriate fluorescein-conjugated secondary antibody at 37 °C for 1 h. The nuclei were stained with Hoechst33258. Images were taken by confocal fluorescence microscopy (SP8, Leica). Optical z-sections were acquired in 0.4 μm steps. Z-stacks were deconvolved in LAS X software (Leica) using default parameters. All presented maximum intensity projections of deconvolved z-stacks were prepared in ImageJ (NIH, Bethesda, USA).

### In vitro culture of blastocysts

All blastocysts were generated by natural mating of *Tubgcp4* heterozygous mice. The morning of the day on which a vaginal plug was detected was designated day 0.5. Blastocysts were collected on E3.5 by flushing the uterus with M2 medium (Sigma-Aldrich) and cultured in ES medium (DMEM, 15% FBS (SH30070.02E, HyClone), 2 mM L-glutamine, 50 units/ml penicillin, 50 µg/ml streptomycin, 55 µM 2-mercaptoethanol (Invitrogen), and 10^3^ units/ml leukemia inhibitory factor (ESG1106, Millipore, Darmstadt, Germany)). Outgrowths were photographed under an inverted microscope (DM IRB, Leica) each day and harvested for genotyping.

### X-ray imaging

Adult mice were anesthetized by intraperitoneal injection of pentobarbital sodium (85 mg/kg body weight). X-ray imaging was performed using a Bruker In-Vivo Xtreme imaging system (Bruker Xtreme BI, Bruker, Madison, USA). The images were captured by a 4 MP back-illuminated cooled CCD and a f-stop 1.1–16 lens (Bruker Molecular Imaging, Billerica, USA) with the following parameters: 1.2 sec exposure time, f-stop 2.0, binning 1 × 1, X-ray filter: 0.4 mm, X-ray energy: 45kVp. Body indexes were measured by Image-pro plus 6.0 (Media Cybernetics, Rockville, USA).

### Mouse electroretinography

Mouse ERG measurement was performed according to previously described procedures with modifications [52]. Mice were dark adapted overnight and anesthetized with a mixture of ketamine (75 mg/kg body weight) and xylazine (5 mg/kg body weight) under dim red light. The pupils were dilated with a single drop of 1% atropine sulfate. A drop of 0.5% proparacaine hydrochloride was applied for corneal anesthesia. A small amount of 2.5% methylcellulose gel was applied to the eye. Mice were placed on the heating pad (37 °C) of a Ganzfeld dome (Roland Q400, Wiesbaden, Germany). A silver loop electrode was placed over the cornea to record the ERGs. Needle reference and ground electrodes were placed in the cheek and tail, respectively. All stimuli were presented in the Ganzfeld dome. Light was spectrally filtered with a 500-nm interference filter. The intensities of flashes were −5, −15, −25, and −35 log scotopic candela-sec/m^2^ (cd. s/m^2^). For photopic ERG, mice were recovered at intensities of 30 cd. s/m^2^ for 10 min, and then tested at the intensity of 3.0 cd. s/m^2^. Data were collected and analyzed with Port32.exe.

### HCQ treatment in *Tubgcp4*^+/−^ mice

Mice were given treatment at the age of 6 months. For hydroxychloroquine (HCQ; H1306, Tokyo Chemical Industry, Japan) treatment [30], HCQ was administered in the drinking water at a concentration of 1.2 mg/ml. In the control group, mice were raised at the same condition except no HCQ administered.

### Electron microscopy

Electron microscopy analysis was performed as described previously [53]. Briefly, eye samples were enucleated, and the anterior segment and the lens were removed. Eye cups were fixed in fixative buffer (4% paraformaldehyde and 3% glutaraldehyde for 4 h in 0.1 M sodium-phosphate buffer, pH 7.4) at 4 °C for at least 24 h, and post-fixed in 1% osmium tetroxide for 2 h at 4 °C. After a stepwise ethanol and acetone dehydration and infiltration with Spurr’s epoxy resin, the samples were embedded and polymerized in Spurr’s epoxy resin at 60 °C for 48 h. Then, the samples were sectioned at a thickness of 70 nm using an ultramicrotome (EM UC7, Leica). The sections were contrasted with 5% uranyl acetate and lead citrate and examined under a transmission electron microscope (Tecnai G^2^ 20, FEI, Oregon, USA).

### Sucrose density gradient centrifugation analysis

Eye samples were enucleated, and retinas were dissected and homogenized at 4 °C in 0.2 ml of HEPES buffer (50 mM HEPES at pH 7.5, 150 mM NaCl, 1 mM MgCl_2_, 1 mM EGTA, and 0.5% NP-40 with protease inhibitor cocktail (04693159001, Roche Applied Science)). After centrifugation for 10 min at 12,000 × *g* at 4 °C, the supernatant was loaded onto a 3.3 mL 5–40% continuous sucrose gradient in HEPES buffer without 0.5% NP-40. The gradient was then centrifuged in a RPS56T-swing rotor (Hitachi, Tokyo, Japan) for 4 h at 314,000 × *g* at 4 °C. Fractions were collected from top to bottom (14 fractions) and analyzed by western blotting.

### Statistical analysis

All data are presented as the means ± standard deviation. Statistical comparisons were made using Student’s *t*-test when comparing two groups. One-way or two-way analysis of variance (ANOVA) with Bonferroni posttest was performed for comparisons among more than two groups. Statistical analysis was performed using the GraphPad Prism 5 software package (GraphPad Software, La Jolla, USA). For all analysis, a p-value < 0.05 was considered to be statistically significant.

### Ethics statement

All animal experiments and methods were performed in accordance with the relevant approved guidelines and regulations, as well as under the approval of the Ethics Committee of Wuhan University.

## Supplementary information

SUPPLEMENTAL MATERIAL
