# Supplementary material for: Haploinsufficiency of GCP4 induces autophagy and leads to photoreceptor degeneration due to defective spindle assembly in retina
Source: Cell Death Differ. 2019 Jun 17;27(2):556–72. doi: 10.1038/s41418-019-0371-0 (PMC7206048; doi:10.1038/s41418-019-0371-0)
Supplement: Supplementary file 1 — SUPPLEMENTAL MATERIAL [file 41418_2019_371_MOESM1_ESM.pdf]

Supplementary information for

Haploinsufficiency of GCP4 induces autophagy and leads to photoreceptor degeneration due to defective spindle assembly in retina

Running title: Retinopathy and GCP4 mutation

Zhigang Li,[1] Huirong Li,[2] Xu Xu,[1] Lingling Wang,[1] Bo Liu,[2] Weixin Zheng,[1] Lili Lian,[2] Ying Song, [1] Xizhong Xia,[1] Ling Hou,[2] Hanhua Cheng[1,*], Rongjia Zhou[1,*]

[1]Hubei Key Laboratory of Cell Homeostasis, College of Life Sciences, Wuhan University, Wuhan 430072, China

[2]State Key Laboratory of Opthalmology, Optometry and Vision Science, Wenzhou Medical University, Wenzhou, 325003, China

*Corresponding authors: Professors Rongjia Zhou and Hanhua Cheng, College of Life Sciences, Wuhan University, Wuhan 430072, P. R. China, Fax: 0086-27-68756253, E-mail: rjzhou@whu.edu.cn, hhcheng@whu.edu.cn

Including 10 figures and 1 table.

Materials and methods

Real-time PCR

TRIzol (15596-026, Invitrogen, Carlsbad, USA) was used to isolate total RNA, which was reverse transcribed using a poly (T)18 primer and reverse transcriptase (M1701, Promega, Madison, USA). Platinum SYBR Green qPCR Super Mix-UDG (D01010A, Invitrogen) was used for real-time PCR amplification of *Tubgcp4* in a StepOne real-time PCR system (Applied Biosystems, Carlsbad, California).

Lentivirus generation and infection

To generate lentivirus as described previously [1], briefly, HEK 293T cells in 10 cm-plates were transfected with lentiCRISPRv2-GCP4-gRNA and lentiviral packaging vectors (pRSV-Rev (12253, Addgene), pMD2.G (12259, Addgene) and pCMV-VSV-G (8454, Addgene)) using Lipofectamine 2000 according to the manufacturer's instructions. After incubation for 48 hours, the supernatants were filtered through 0.45 μm filters and used directly to infect MEF cells. Puromycin (P0108-25MG, Ekear, Shanghai, China) was used to screen cells with lentiCRISPRv2.

Detection of rd8 mutation by PCR

DNA samples isolated from mouse tail biopsies were amplified separately for wild type allele and mutant rd8 allele using primers listed in **Table S1**. The wild type allele yielded a 220 bp product and rd8 allele yielded a 244 bp product.

RPE flat mounts

RPE flat mounts were performed as described previously [2]. The samples of posterior cup of eyes were prepared from enucleated eyes and fixed in methanol solution 10 minutes at 4°C. Retinae of the isolated eyecups were teased free from the RPE and removed, and the RPE/choroid/sclera tissues were cut into a four-leaf clover shape. RPE flat mounts were blocked with 1% BSA for 30 minutes at room temperature. The samples were incubated at 4°C overnight with anti-Rhodopsin (MAB5316, Millipore, Darmstadt, Germany) and anti-ZO1 (40-2200, Invitrogen) followed by Alexa Fluor 594-conjugated donkey anti-Mouse (1:300, Invitrogen) and Alexa Fluor 488-conjugated goat anti-Rabbit IgG (H+L) secondary antibody

(R37116, Invitrogen) respectively. Nuclei were stained with Hoechst33258 (861405, Sigma-Aldrich, St Louis, USA). Images were taken by confocal fluorescence microscopy (SP8, Leica, Wetzlar, Germany).

1. Yuan J, Zhang Y, Sheng Y, Fu X, Cheng H, Zhou R. MYBL2 guides autophagy suppressor VDAC2 in the developing ovary to inhibit autophagy through a complex of VDAC2-BECN1-BCL2L1 in mammals. *Autophagy* 2015; **11**(7)**:** 1081-1098.

2. Kim JY, Zhao H, Martinez J, Doggett TA, Kolesnikov AV, Tang PH*, et al.* Noncanonical autophagy promotes the visual cycle. *Cell* 2013; **154**(2)**:** 365-376.

Figure S1

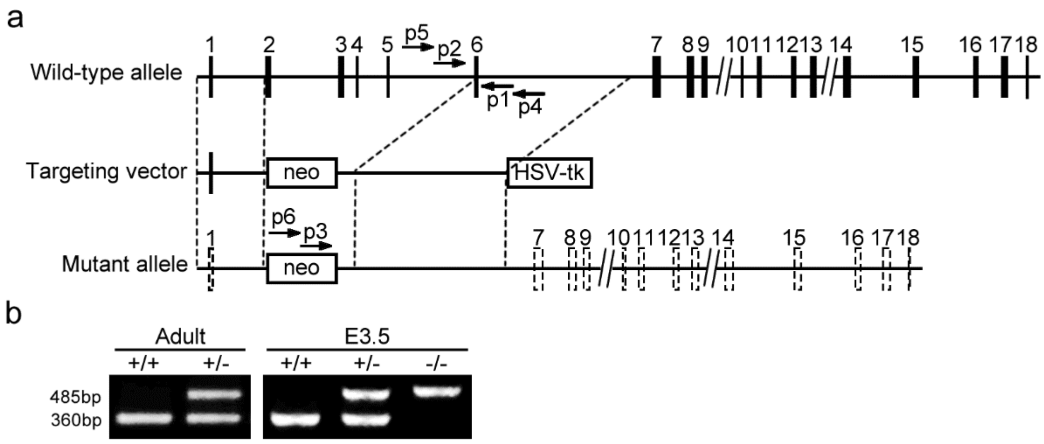

Figure S1. Schematic representation of the targeting strategy of *Tubgcp4* and genotyping. (**a**) *Tubgcp4* locus, the targeting vector and the targeted loci. The exons 2-6 were replaced with neomycin resistance gene (*Neo*), which generated exons 2-6 deletion and frameshift after exon 1. The targeting vector contained a herpes simplex virus-thymidine kinase (*HSV-tk*) and *Neo* genes. The arrows indicated the position and direction of primers used for genotyping (p4, p5, p6 for the first-round PCR; p1, p2, p3 for the nest PCR). (**b**) Representative genotyping analysis of adult mice and E3.5 embryos obtained from *Tubgcp4*$^{+/-}$ mating. Genomic DNA was isolated from individual embryos or tails, and genotypes were determined by PCR using primers described in A. For E3.5 embryo genotyping, after first-round PCR using primers p4, p5 and p6, the PCR products were used as templet of the nest PCR. In the nest PCR, p1 and p2 generated a wild-type band of 360 bp and p1 and p3 produced a heterozygous band of 485bp. For adult genotyping, no nest PCR, but the same sets of primers were used.

Figure S2

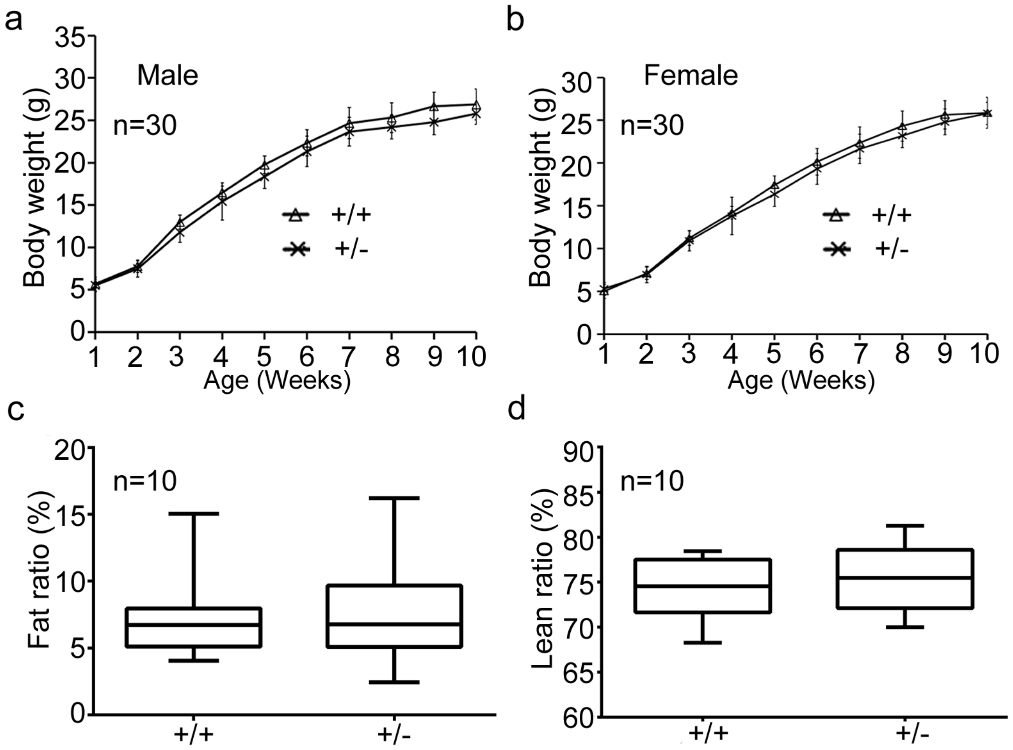

Figure S2. Growth rate, body fat and lean mass in heterozygous mice. *Tubgcp4*$^{+/-}$ mice were viable, fertile and morphologically no different from the wild-type littermates. (**a, b**) The growth rate of littermates was no significantly different in male (**a**) or female mice (**b**). The weight of *Tubgcp4*$^{+/-}$ and wild-type littermates were weekly monitored from 1 to 10 weeks of age. The data were analyzed using ANOVA followed by Bonferroni posttest (n=30). Data were presented as means± SD. (**c**, **d**) Whole body fat and lean mass were comparable between *Tubgcp4*$^{+/-}$ and wild-type littermates as measured by minispec LF50 body composition analyzer (LF50, Bruker, Germany) (n=10). The data were analyzed using Student's *t*-test (n=30). Data were presented as means± SD.

Figure S3

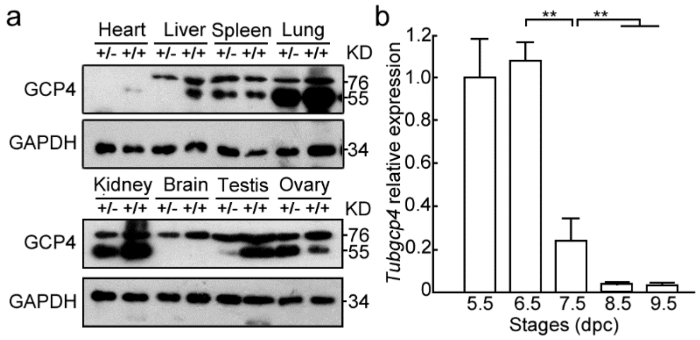

Figure S3. GCP4 expression in adult tissues and embryos. (**a**) Western blot analysis showed that GCP4 widely expressed in adult tissues. The expression level was lower in heart than other tissues. Both bands (76 and 55 KD) were products of two transcripts of *Tubgcp4* as alternative ATG. GAPDH was employed as an internal control. (**b**) Real-time quantitative PCR of *Tubgcp4* in early post-implantation embryos. An expression peak was detected at E6.5. *Actb* was used as an internal control.

Figure S4

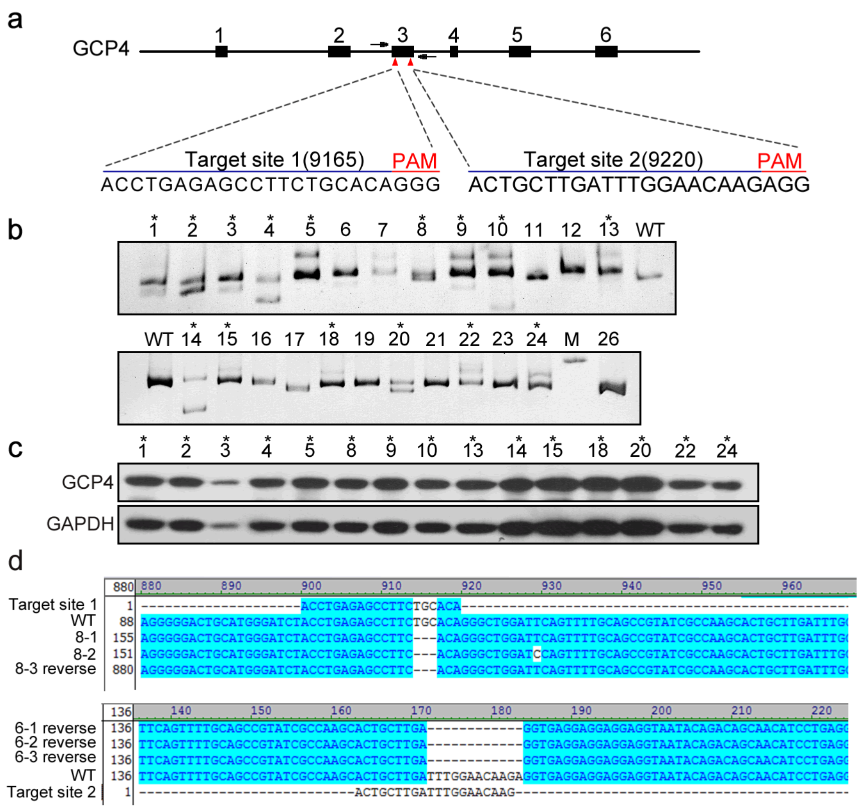

Figure S4. Targeted disruption of *Tubgcp4* using CRISPR/Cas9 system. (**a**) Schematic representation of gRNAs targeting the *Tubgcp4* locus. The coding exons were indicated with black box. Two gRNAs (red arrowheads) were designed to target open reading frame in exon 3. PAM, protospacer adjacent motif, were highlighted in red. Arrows indicated primer positions for genotyping of progeny. (**b**) PCR analysis of genomic DNA isolated from single MEF clone. The numbers indicated different clones. Asterisks indicated potential KO clones prepared for western blot analysis. Primer sequences and PCR conditions were listed in Table S1. WT, wild-type MEF; M, DL2000 marker. (**c**) Western blot analysis of GCP4 expression in the MEF clones in panel **b**. GAPDH was used as an internal control. (**d**) Nucleotide sequence alignments of *Tubgcp4* mutant alleles of #6 and #8 clones with WT sequence. The deleted sequences were indicated in dash lines. The targeting generates deletions of multiples of 3 bases and did not lead to frameshift mutation in these clones.

Figure S5

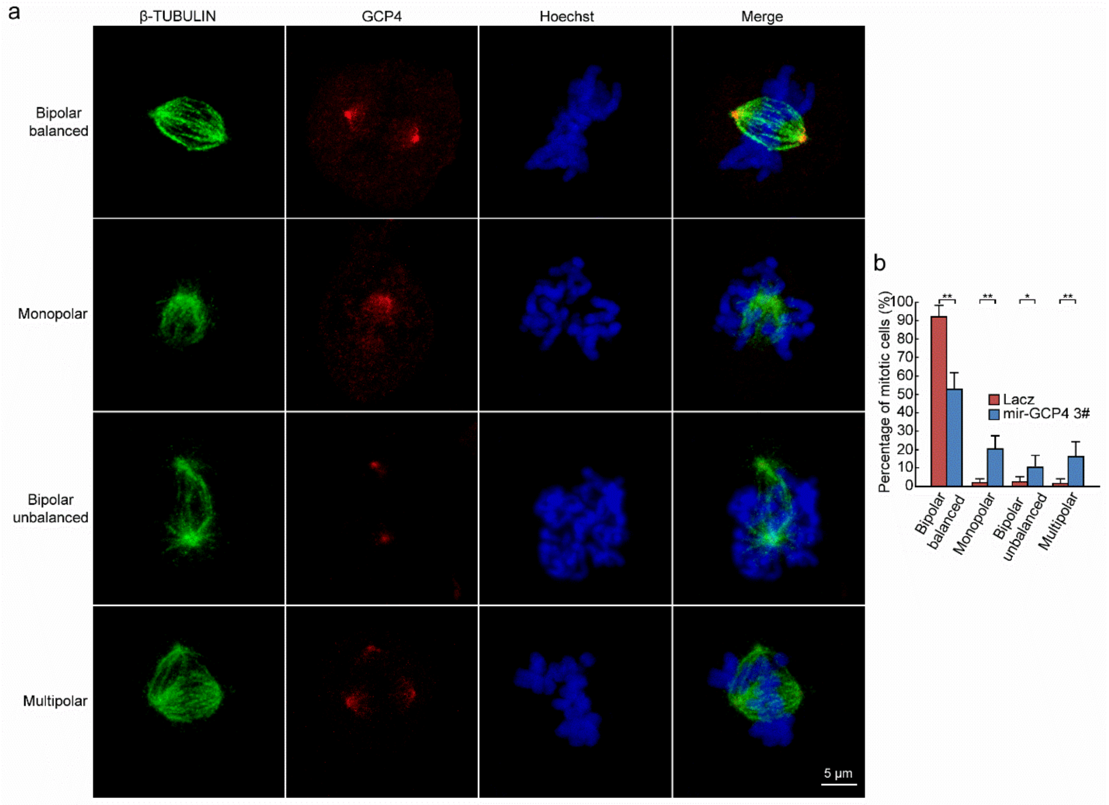

Figure S5. GCP4 knockdown disturbs mitotic spindle formation using mir-GCP4 3#. (**a**) Representative images of impaired mitotic spindles. Endogenous GCP4 and β-TUBULIN were examined by indirect immunofluorescence using anti-GCP4 (red) and anti-β-TUBULIN (green) antibodies. Images were taken by confocal microscopy. The nuclei were stained with Hoechst reagent. Arrowhead indicates a potential spindle pole; Arrows indicate spindle poles. Bipolar balanced, cells with a broad-based bipolar spindle; Monopolar, cells with only one spindle pole; Bipolar unbalanced, cells with an unequal bipolar spindle; Multipolar, cells with ⩾ 3 spindle poles. Scale bar: 5 μm. (**b**) Quantification of the mitotic spindle types in panel **a**. Data were represented as means± SD (n = 3 experiments, with 50 cells/experiment). p values were calculated by two-tailed t test: *$p \leq 0.05$; **$p \leq 0.01$.

Figure S6

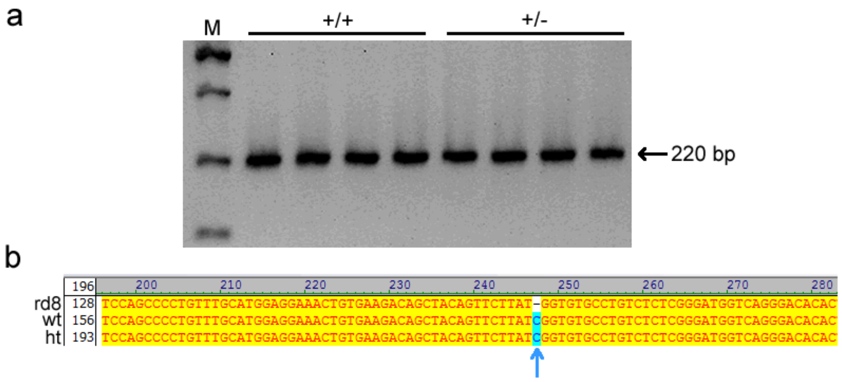

Figure S6. *Tubgcp4* knockout mice were free of background mutation of rd8. (**a**) A specific wild-type allele (220 bp) was observed and no rd8 mutation allele was detected. (**b**) Sequence of Crb1 gene showed no single base deletion at the expected position in the aligned sequences. rd8, rd8 mutation sequence; wt, DNA sequence of wild type mice; ht, DNA sequence of heterozygotes.

Figure S7

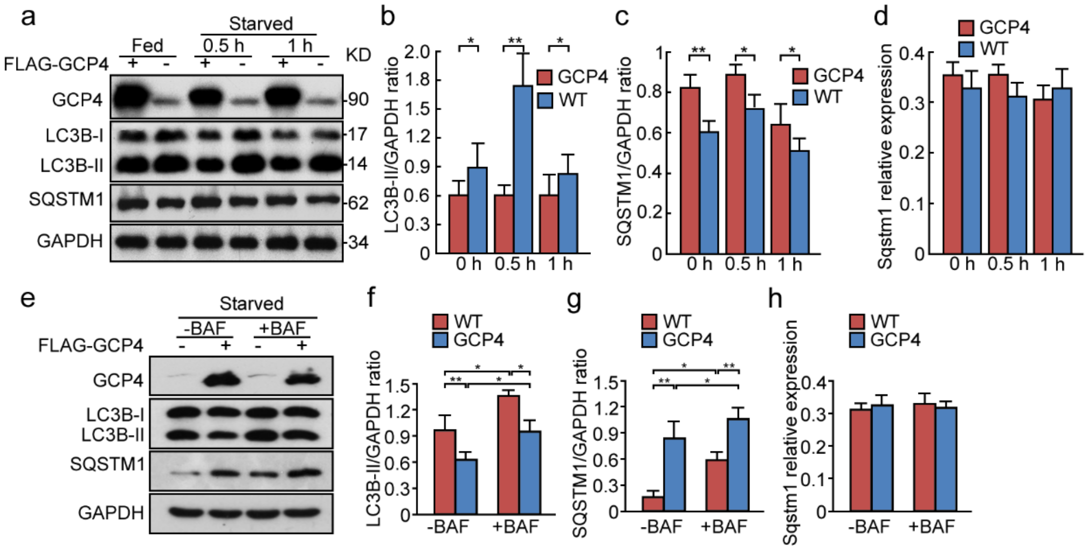

Figure S7. GCP4 over-expression inhibits autophagy. (**a**) GCP4 over-expression downregulated LC3B-II level under both starvation and fed conditions. HEK293T cells were transfected with equal amounts of 3xFlag-GCP4 (+) or vector pCMV-tag2B (-) and cultured in the EBSS medium for 0, 0.5 and 1 h respectively. Cell lysates were analyzed by immunoblotting with the indicated antibodies. GAPDH was used as an endogenous control. (**b**, **c**) Quantification of LC3B-II (**b**) and SQSTM1 (**c**) expression levels in panel **a**. The results were representative of 3 independent experiments and represented as the means ± SD. The data were analyzed by Student's *t*-test. *$p < 0.05$; **$p < 0.01$. (**d**) Real-time quantitative PCR of *Sqstm1* in the cells treated as in panel **a**. *β-actin* was used as an internal control. The results were representative of 3 independent experiments and represented as the means ± SD. The data were analyzed by Student's *t*-test. (**e-h**) GCP4 over-expression showed accumulation of both LC3B-II and SQSTM1 protein levels when Bafilomycin A1 treated compared with non-treated cells. The same cells in panel **a** were cultured in EBSS with or without BAF (100 nM) for 4 h. The cell lysates were analyzed by immunoblotting with antibodies as indicated. GAPDH was used as an endogenous control. (**f**, **g**) Quantification of LC3B-II (**f**) and SQSTM1 (**g**) expression levels in panel **e**. The results were representative of 3 independent experiments and represented as the means ± SD. The data were analyzed by one-way ANOVA followed by Bonferroni posttest. *p<

0.05; $^{**}p < 0.01$. (**h**) Real-time quantitative PCR of *Sqstm1* in the cells treated as in panel e. *β-actin* was used as an internal control. The results were representative of 3 independent experiments and represented as the means ± SD. The data were analyzed by one-way ANOVA followed by Bonferroni posttest.

Figure S8

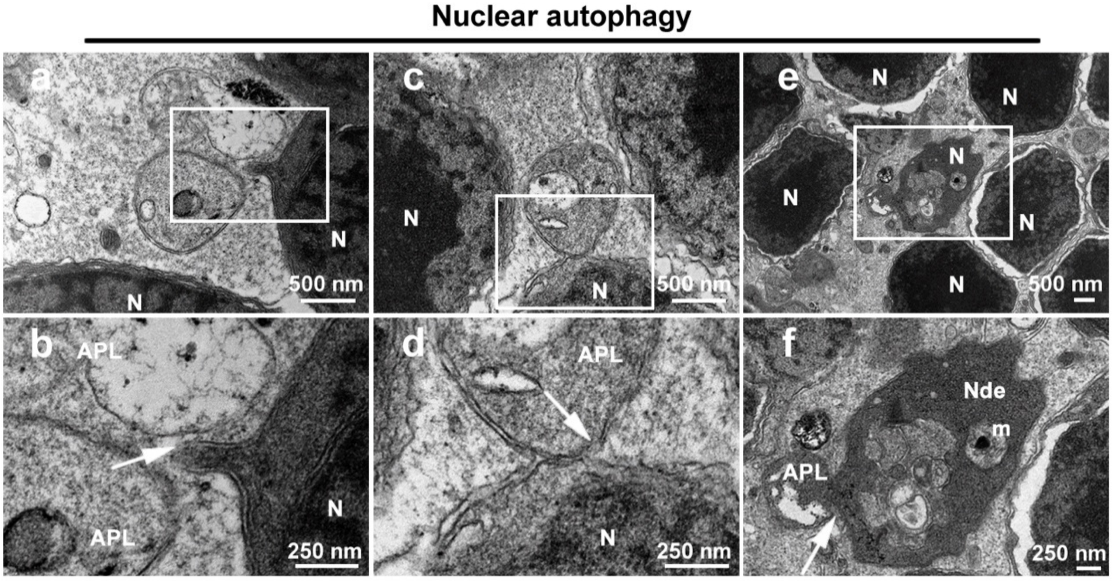

Figure S8. Representative images of nuclear autophagosomes in photoreceptor cells. The nuclear membrane protrudes (**a**, **b**), contacts (**c**, **d**) and fuses with autolysosomes (**e**, **f**). The images in the white squares in panels **a**, **c,** and **e** were enlarged and showed in panels **b**, **d,** and **f**, respectively. Arrows indicated the contacted area of the autolysosomes with nuclear membrane. N, the nuclei; APL, autolysosomes; m, mitochondria; Nde, degrading nucleus. Scale bars are indicated in each image.

Figure S9

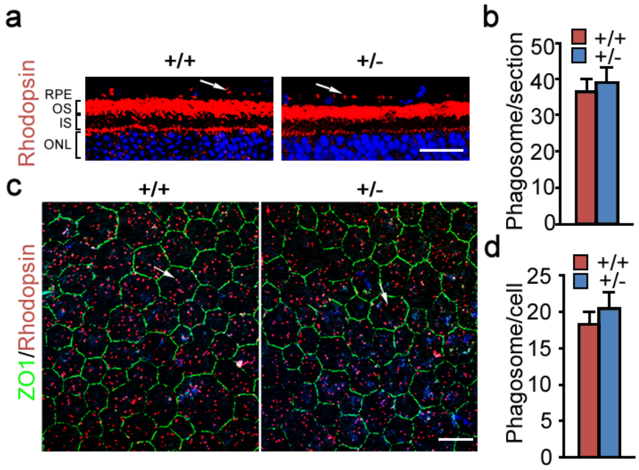

Figure S9. Phagocytic ability of RPE. (**a**) Immunofluorescence analysis of the Rhodopsin puncta in the heterozygous and wild-type retinas using anti-Rhodopsin antibody. Rhodopsin puncta were mainly located in the outer segment of photoreceptor cells in both heterozygous and wild-type retinas. Some Rhodopsin puncta were also observed in the RPE cells (white arrows). The nuclei were stained with Hoechst reagent. RPE, retinal pigment epithelium; OS, outer segment; IS, inner segment; ONL, outer nuclear layer; Scale bar: 25 μm (**b**) Statistic analysis of Rhodopsin puncta per section using Student's *t*-test. Three retinas from three mice were sectioned and three sections were counted per retina. Data were represented as means± SD. **(c)** Immunofluorescence analysis of the Rhodopsin puncta (white arrows) in the heterozygous and wild-type RPE flat mounts using anti-Rhodopsin and anti-ZO1 antibody on RPE flat mounts. (**d**) Statistic analysis of Rhodopsin puncta per cell using Student's *t*-test. Three retinas from three mice were sectioned and three sections were counted per retina. Data were represented as means± SD.

Figure S10

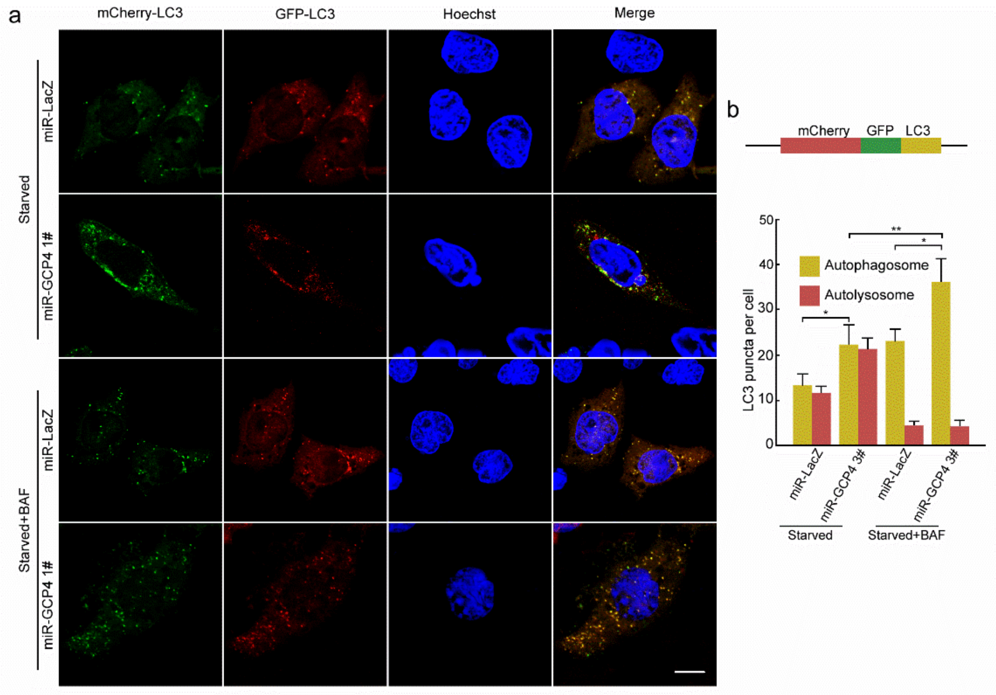

Figure S10. Autophagy flux associated with GCP4 using stable miR-*Gcp4*-3# cell line. (**a**) Detection of autophagy flux using fluorescent confocal microscopy. Stable miR-*Gcp4*-3# and miR-*LacZ* COS-7 cells were transfected with a tandem expression vector mCherry-GFP-LC3 and cultured in EBSS medium for 2 h. In the BAF+ EBSS group, the cells were treated with BAF for 4 h to suppress the fusion between autophagosome and lysosome. Yellow or green puncta indicated autophagosomes, while red puncta include autophagosomes and autolysosomes, because GFP protein is sensitive and attenuated in an acidic environment of autolysosome. Scale bar: 10 μm. (**b**) The tandem structure of mCherry-GFP-LC3 and statistical analysis of LC3 puncta per cell (*n* = 3 experiments, with 40 cells/experiment). Data were represented as means± SD. Two-way ANOVA followed by Bonferroni posttest was used for statistical analysis. *$p< 0.05$; **$p< 0.01$.

Table S1. The primers used in the study

Primer names	Primer sequences (5'-3')	PCR conditions
ES-5'arm PCR		
Tubgcp4-5P	AGAGTAGGCATCTCCAAGCAAGC	94 °C, 45 s; 64 °C, 2 min; 34 cycles
neo-R	CTGAGCCCAGAAAGCGAAGGA	
ES-3'arm PCR		
neo-F	CCTCCCCCGTGCCTTCCTTGAC	94 °C, 45 s; 68 °C, 2 min; 34 cycles
Tubgcp4-3P	CCCTGGAGTAGAAGAGATGGCTTGGTGG	
Genotyping		
GT-f1	ATTTCTCGATGGCCTTTGCTTCAG	94 °C, 30 s; 60 °C, 30 s; 72 °C, 30 s; 35 cycles
GT-f2	TGATATTGCTGAAGAGCTTGGC	
GT-r	TGACTCCCTCCCCTATTCCCAA	
Genotyping-nest		
GT-nest-f1	CCCTACTAGCCTTCATGATGACCT	94 °C, 30 s; 60 °C, 30 s; 72 °C, 30 s; 35 cycles
GT-nest-f2	TCGCCTTCTATCGCCTTCTTGAC	
GT-nest-r	CCCAATACTTACGGAAGCCAGGA	
Rd8 detection		94 °C, 30 s; 65 °C, 30 s; 72 °C, 30 s; 35 cycles
mCrb1-f1	GTGAAGACAGCTACAGTTCTGATC	
mCrb1-f2	GCCCCTGTTTGCATGGAGGAAACTTGGAAGACAGCTACA GTTCTTCTG	
mCrb1-r	GCCCCATTTGCACACTGATGAC	
Tubgcp4-RT-PCR		
Tubgcp4-RT-f	GCAGCCTTATCGCCAAGCA	94 °C, 30 s; 58 °C, 30 s; 72 °C, 30 s;

		40 cycles
Tubgcp4-RT-r	AGCTGTTTATACATGACCCCA	
Actin-RT-PCR		
Actin-RT-f	GTGGTGGTGAAGCTGTAGCC	94 °C, 30 s; 58 °C, 30 s; 72 °C, 30 s; 40 cycles
Actin-RT -r	ACTGTGCCCATCTACGAGGG	
Tubgcp4-CDS		
Tubgcp4-CDS-f	ATAGAATTCATGATTCACGAACTGCTCTT	94°C, 30 s; 64°C - 1°C, 30 s; 72°C, 2 min, 10 cycles; 94°C, 30 s; 60°C, 30 s; 72°C, 2 min, 25 cycles
Tubgcp4-CDS-r	ATACTCGAGCATCCCAAAACTGCCCAGAG	
Cmyc-Atg7		
Cmyc-Atg7-f	ATAGAATTCATGGCGGCAGCTACGGGGGA	94°C, 30 s; 60°C - 1°C, 30 s; 72°C, 2min, 10 cycles; 94°C, 30 s; 55°C, 30 s; 72°C, 2 min, 25 cycles
Cmyc-Atg7-r	ATAGTCGACTCAGATGGTCTCATCATCGC	
Cmyc-Atg7-NTD		
Cmyc-Atg7-NTD-f	ATAGAATTCATGGCGGCAGCTACGGGGGA	94°C, 30 s; 60°C - 1°C, 30 s; 72°C, 1min, 10 cycles; 94°C, 30 s; 55°C, 30 s; 72°C, 1 min, 25 cycles
Cmyc-Atg7-NTD-r	ATAGTCGACACTGAGGTTCACCATCCTTG	
Cmyc-Atg7-CTD		
Cmyc-Atg7-CTD-f	ATAGAATTCGAATGTATGGACCCTAAAAGGTTA	94°C, 30 s; 60°C - 1°C, 30 s; 72°C, 1min, 10 cycles;

Cmyc-Atg7-CTD-r	ATAGTCGACTCAGATGGTCTCATCATCGC	94°C, 30 s; 55°C, 30 s; 72°C, 1 min, 25 cycles
miR-LacZ		
miR-LacZ-f	CACTGACTGACGACTACACAAATCAG<br>CGATTTCAGGACACAAGGCCTGTTAC	94°C, 30 s; 65°C - 1°C, 30 s; 72°C, 5 min, 10 cycles; 94°C, 30 s; 60°C, 30 s; 72°C, 5 min, 25 cycles
miR-LacZ-r	GCCAAAACGACTACACAAATCAGCG<br>ATTTCAGCATACAGCCTTCAGCAAG	
miR-Tubgcp4-1#		
miR-Tubgcp4-1#-f	CACTGACTGACGCCAAGGTGTTACA<br>TGGAACAGGACACAAGGCCTGTTAC	94°C, 30 s; 65°C - 1°C, 30 s; 72°C, 5 min, 10 cycles; 94°C, 30 s; 60°C, 30 s; 72°C, 5 min, 25 cycles
miR-Tubgcp4-1#-r	GCCAAAACGCCAAGGTGGGTTACAT<br>GGAACAGCATACAGCCTTCAGCAAG	
miR-Tubgcp4-2#		
miR-Tubgcp4-2#-f	CACTGACTGACCTATATTCTTCACT<br>GAGTTCAGGACACAAGGCCTGTTAC	94°C, 30 s; 65°C - 1°C, 30 s; 72°C, 5 min, 10 cycles; 94°C, 30 s; 60°C, 30 s; 72°C, 5 min, 25 cycles
miR-Tubgcp4-2#-r	GCCAAAACCTATATTCGCTTCACTG<br>AGTTCAGCATACAGCCTTCAGCAAG	
miR-Tubgcp4-3#		
miR-Tubgcp4-3#-f	CACTGACTGACAATTTGCTCTCAAT<br>CCTTTCAGGACACAAGGCCTGTTAC	94°C, 30 s; 65°C - 1°C, 30 s; 72°C, 5 min, 10 cycles; 94°C, 30 s; 60°C, 30 s; 72°C, 5 min, 25 cycles
miR-Tubgcp4-3#-r	GCCAAAACAATTTGCTGGCTCAATC<br>CTTTCAGCATACAGCCTTCAGCAAG	

Flag-ATG3		
Flag-ATG3-f	ATAGAATTCATGCAGAATGTGATCAACACG	94°C, 30 s; 65°C -1°C, 30 s; 72°C, 1 min, 10 cycles; 94°C, 30 s; 60°C, 30 s; 72°C, 1 min, 25 cycles
Flag-ATG3-r	ATACTCGAGCTACATTGTGAAGTGTCTTGTGT	
LentiCRISPRv2-GCP4-1#		
LentiCRISPRv2-GCP4-1#-f	CACCGACCTGAGAGCCTTCTGCACA	
LentiCRISPRv2-GCP4-1#-r	AAACTGTGCAGAAGGCTCTCAGGTC	
LentiCRISPRv2-GCP4-2#		
LentiCRISPRv2-GCP4-2#-f	CACCGACTGCTTGATTTGGAACAAG	
LentiCRISPRv2-GCP4-2#-r	AAACCTTGTTCCAAATCAAGCAGTC	
LentiCRISPRv2-GCP4-ID		
LentiCRISPRv2--GCP4-ID-f	ACGGTATCTTCTCGGGTTGC	
LentiCRISPRv2--GCP4-ID-r	TGACCTTCTCAAACGCCACT	
Sqstm1-qPCR-mouse		
Sqstm1-qPCR-f	AGGAAGCTGCCCTATACCCACA	94 °C, 30 s; 61°C, 30 s; 72 °C, 30 s; 40 cycles
Sqstm1-qPCR-r	CCAGCCGCCTTCATCCGAGA	
SQSTM1-qPCR-homo		
SQSTM1-qPCR-f	AATCAGCTTCTGGTCCATCG	94 °C, 30 s; 58°C, 30 s; 72 °C, 30 s; 40 cycles
SQSTM1-qPCR-r	TTCTTTTCCCTCCGTGCTC	
ACTIN-qPCR-homo		
ACTIN-qPCR-f	AGAGCTACGAGCTGCCTGAC	94 °C, 30 s; 58°C, 30 s; 72 °C, 30 s;

		40 cycles
ACTIN-qPCR-r	AGCACTGTGTTGGCGTACAG	
